# The genetic interplay between body mass index, breast size and breast cancer risk: a Mendelian randomization analysis

**DOI:** 10.1093/ije/dyz124

**Published:** 2019-06-26

**Authors:** Brandon Nick Sern Ooi, Huiwen Loh, Peh Joo Ho, Roger L Milne, Graham Giles, Chi Gao, Peter Kraft, Esther M John, Anthony Swerdlow, Hermann Brenner, Anna H Wu, Christopher Haiman, D Gareth Evans, Wei Zheng, Peter A Fasching, Jose Esteban Castelao, Ava Kwong, Xia Shen, Kamila Czene, Per Hall, Alison Dunning, Douglas Easton, Mikael Hartman, Jingmei Li

**Affiliations:** 1 Human Genetics, Genome Institute of Singapore, Singapore, Singapore; 2 Cancer Epidemiology Division, Cancer Council Victoria, Melbourne, VIC, Australia; 3 Program in Genetic Epidemiology and Statistical Genetics, Harvard T.H. Chan School of Public Health Boston, USA; 4 Department of Medicine and Stanford Cancer Institute, Stanford University School of Medicine, Stanford, CA, USA; 5 Division of Genetics and Epidemiology and Division of Breast Cancer Research, Institute of Cancer Research, London UK; 6 Division of Clinical Epidemiology and Aging Research, German Cancer Research Center (DKFZ) Heidelberg, Germany; 7 Division of Preventive Oncology, German Cancer Research Center (DKFZ) and National Center for Tumor Diseases (NCT), Heidelberg, Germany; 8 German Cancer Consortium (DKTK), German Cancer Research Center (DKFZ), Heidelberg, Germany; 9 Department of Preventive Medicine, Keck School of Medicine, University of Southern California, Los Angeles, CA, USA; 10 Genomic Medicine, Division of Evolution & Genomic Sciences, The University of Manchester Manchester, UK; 11 Division of Epidemiology, Department of Medicine, Vanderbilt Epidemiology Center, Vanderbilt University Medical Centre, Vanderbilt University Nashville, USA; 12 Department of Gynecology and Obstetrics, University Hospital Erlangen, Comprehensive Cancer Center Erlangen-EMN, Friedrich-Alexander University Erlangen-Nuremberg, Germany; 13 Oncology and Genetics Unit, Instituto de Investigacion Sanitaria Galicia Sur (IISGS), Xerencia de Xestion Integrada de Vigo-SERGAS, Vigo, Spain; 14 Department of Surgery, The University of Hong Kong Pok Fu Lam, Hong Kong; 15 Department of Medical Epidemiology and Biostatistics, Karolinska Insititute Stockholm, Sweden; 16 Biostatistics Group, State Key Laboratory of Biocontrol, School of Life Sciences, Sun Yat-sen University, Guangzhou, China; 17 Center for Global Health Research, Usher Institute of Population Health Sciences and Informatics, University of Edinburgh, Edinburgh, UK; 18 Centre for Cancer Genetic Epidemiology, Department of Oncology, University of Cambridge, Cambridge, UK; 19 Department of Surgery, Yong Loo Lin School of Medicine, National University of Singapore, Singapore, Singapore

**Keywords:** Breast size, breast cancer risk, body mass index, Mendelian randomization, LDSC regression, genetic epidemiology, genetic correlation

## Abstract

**Background:**

Evidence linking breast size to breast cancer risk has been inconsistent, and its interpretation is often hampered by confounding factors such as body mass index (BMI). Here, we used linkage disequilibrium score regression and two-sample Mendelian randomization (MR) to examine the genetic associations between BMI, breast size and breast cancer risk.

**Methods:**

Summary-level genotype data from 23andMe, Inc (breast size, *n *=* *33 790), the Breast Cancer Association Consortium (breast cancer risk, *n *=* *228 951) and the Genetic Investigation of ANthropometric Traits (BMI, *n *=* *183 507) were used for our analyses. In assessing causal relationships, four complementary MR techniques [inverse variance weighted (IVW), weighted median, weighted mode and MR-Egger regression] were used to test the robustness of the results.

**Results:**

The genetic correlation (*rg*) estimated between BMI and breast size was high (*rg *=* *0.50, *P* = 3.89x10^−43^). All MR methods provided consistent evidence that higher genetically predicted BMI was associated with larger breast size [odds ratio (OR_IVW_): 2.06 (1.80–2.35), *P* = 1.38x10^−26^] and lower overall breast cancer risk [OR_IVW_: 0.81 (0.74–0.89), *P* = 9.44x10^−6^]. No evidence of a relationship between genetically predicted breast size and breast cancer risk was found except when using the weighted median and weighted mode methods, and only with oestrogen receptor (ER)-negative risk. There was no evidence of reverse causality in any of the analyses conducted (*P* > 0.050).

**Conclusion:**

Our findings indicate a potential positive causal association between BMI and breast size and a potential negative causal association between BMI and breast cancer risk. We found no clear evidence for a direct relationship between breast size and breast cancer risk.

**Key Messages**

- The relationships between breast size, breast cancer risk and body mass index (BMI) were examined using summary-level genotype data from three publicly available sources.
- Genetic associations between these three factors were estimated using (i)genetic variants across the entire genome by linkage disequilibrium score segression, and (ii) only significantly associated variants as instrumental variables in two-sample Mendelian randomization analysis.
- Women with a genetic predisposition to high BMI are likely to have a larger breast size and a lower risk of breast cancer.
- There is no clear evidence for a direct and unmediated association between breast size and breast cancer risk.

## Introduction

Observational studies suggest that breast size is related to breast cancer risk, although the evidence is not consistent (reviewed in^1^). Some studies have shown that larger breast size may be associated with greater breast cancer risk^2–4^ but others have reported an inverse relationship.^5^ At the genetic level, Eriksson *et al.*^6^ found that genetic variants associated with bigger bra cup size were also associated with increased breast cancer risk. However, the link between breast size and breast cancer risk becomes less clear when other factors, such as body mass index (BMI), are considered at the same time.^2^^,^^5^

On the other hand, BMI has established relationships with both breast size and breast cancer risk. Generally, higher BMI is associated with larger breast size.^7–9^ In a twin study, the overlap in genetic heritability for BMI and breast size was estimated to be ∼33%.^10^ However, the link between BMI and breast cancer risk is dependent on menopausal status. An inverse relationship is commonly observed between BMI and premenopausal breast cancer; a positive relationship is commonly observed between BMI and postmenopausal breast cancer.^11–14^ In terms of cancer subtype, the associations are reported to be stronger for hormone receptor-positive breast cancer in both pre- and post-menopausal women.^14–16^ However, several Mendelian randomization (MR) analyses showed that BMI predicted by genome-wide association studies (GWAS)-identified variants was inversely associated with the risk of both pre- and post-menopausal breast cancer.^17–19^

**Figure 1.**
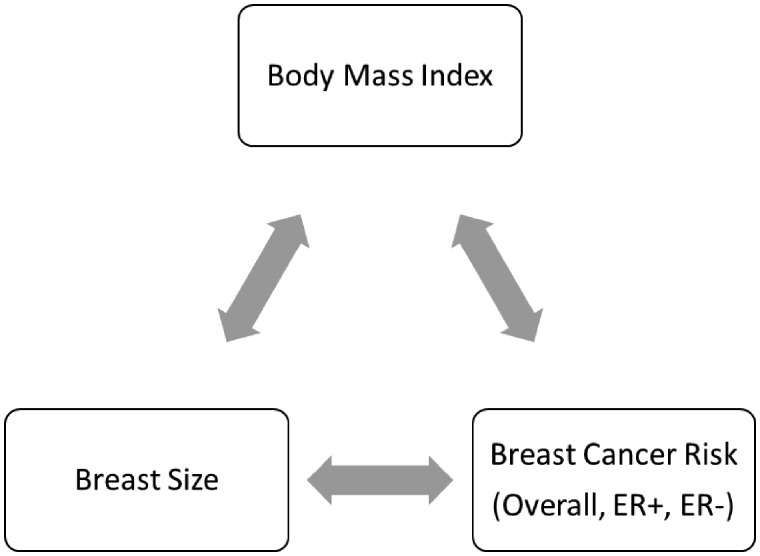
Relationships between the three traits examined in this study.

In this study, we examined the genetic interplay between BMI, breast size and breast cancer risk (Figure 1). Specifically, we (i) used linkage disequilibrium score regression (LDSC)^20^ to estimate genetic correlation by considering genetic variants across the entire genome, and (ii) assessed causality using only significantly associated genetic variants as instruments in two-sample MR analyses.^21^ Both these methods have been selected for their respective strengths—LDSC for the case of complex traits where thousands of variants can have small effects, and MR for its ability to infer causality given that certain assumptions hold.

## Methods

### Summary statistics for BMI, breast size and breast cancer risk

Beta coefficients and standard errors for BMI, breast size and breast cancer risk for women of European ancestry were obtained from three publicly available sources: Genetic Investigation of ANthropometric Traits (GIANT),^22^ 23andMe (version 4.1)^23^ and the Breast Cancer Association Consortium (BCAC).^24–26^ The genetic associations with BMI are in standard deviation (SD) units whereas breast size was coded from 0 to 9, corresponding to an increase in units of bra cup size: smaller than AAA, AAA, AA, A, B, C, D, DD, DDD, and larger than DDD. Breast cancer was coded as a binary phenotype for overall, oestrogen receptor (ER)-positive and ER-negative breast cancer. Further details are provided in Supplementary Table 1, available as Supplementary data at *IJE* online.

### Cross-trait genetic correlation estimated using LDSC

LDSC, which relies on the adjustment for linkage between single nucleotide polymorphisms (SNPs), was used to estimate cross-trait genetic correlations between BMI, breast size and breast cancer risk (overall, ER-positive, and ER-negative).^20^ LDSC estimates genetic correlation by considering the effects of all SNPs, including those that do not reach genome-wide significance. SNPs for BMI, breast size and breast cancer risk, respectively, were merged with HapMap3 SNPs and duplicate reference SNP cluster ids (rsids) were removed using the software provided at https://github.com/bulik/ldsc (Supplementary Table 1, available as Supplementary data at *IJE* online). Linkage disequilibrium scores provided by the software’s creators, based on the 1000 Genome Project’s European samples, were downloaded from the same website and cross-trait genetic correlation, *rg*, was computed from common SNPs between each pair of traits. After all preprocessing steps, there were 1 053 312 common SNPs for BMI and breast size, 992 833 common SNPs for BMI and all three breast cancer traits, and 1 116 435 common SNPs for breast size and all three breast cancer traits.

### SNP selection

Independent SNPs with genome-wide significant associations for BMI (*n *=* *77, explaining 2.2% of the variance in BMI)^27^ and breast size (*n *=* *7, explaining ∼1.2% of the variance in breast size)^6^ were used as exposure data for MR analyses. For outcome, we included 172 breast cancer SNPs.^24^ After verifying the independence of selected SNPs using the clumping function in the TwoSampleMR package in R,^21^ 77, 7 and 114 SNPs were retained for further analyses, respectively (Supplementary Tables 2–4, available as Supplementary data at *IJE* online). All 77 BMI SNPs were present in the breast size and breast cancer risk datasets. All 7 breast size SNPs were present in the breast cancer risk dataset, but only 4 were present in the BMI dataset. A total of 113 breast cancer SNPs were present in the breast size dataset and 81 SNPs were present in the BMI dataset. Before performing MR analyses, the signs of the beta coefficients for each SNP across all outcome datasets were aligned to the effective alleles of the exposure datasets.

To assess the MR assumptions, the MR-Egger test was used to detect directional pleiotropy of the genetic instruments, where a regression intercept that significantly differs from zero indicates the presence of directional pleiotropy or the InSIDE assumption (INstrument Strength Independent of Direct Effect) was violated (i.e. the associations with other traits that may affect the outcome via pathways independent of the exposure).^28^ For the datasets used in this study, only for the case of BMI (exposure) versus breast cancer risk (outcome) was the *P*-value for the MR-Egger intercept significantly different from zero. The MR-Pleiotropy RESidual Sum and Outlier (MR-PRESSO) package in R was used for pleiotropy and outlier SNPs detection for this dataset.^29^ Six outliers were identified for BMI (exposure) and overall risk (outcome), four outliers for BMI (exposure) and ER-positive risk (outcome) and three outliers for BMI and ER-negative risk (outcome). After removal of these outliers, the MR-Egger regression intercept no longer differed significantly from zero. The MR-PRESSO package was also used to test the other datasets, but the MR results did not significantly differ when outliers identified in these other datasets were removed.

### Mendelian randomization 

MR was first performed using the inverse-variance weighted (IVW) method.^30^ The IVW method assumes that the MR assumptions are satisfied or that all SNPs are valid instruments. As it is difficult to test the validity of this assumption, three additional MR methods were also used to assess the robustness of the result from the primary analysis under alternative assumptions: weighted median function,^31^ weighted mode function^32^ and MR-Egger regression.^28^ According to Hartwig *et al*.,^32^ the weighted median function provides a valid result under the assumption that >50% of the weight in the model comes from SNPs that satisfy the MR assumptions. The weighted mode function is valid if the majority of SNPs with similar individual causal effect estimates are valid instruments even if other SNPs in the model do not meet the requirements for causal inference using MR. MR-Egger regression allows for horizontal pleiotropic effects. As long as this pleiotropy is not correlated with SNP-exposure associations, the beta-coefficient from the MR-Egger analysis is valid even if horizontal pleiotropy exists. Both forward and reverse causality for each pair of variables were investigated. The above statistical analyses were performed using TwoSampleMR package in R.^21^

## Results

### BMI is genetically correlated with breast size

**Figure 2.**
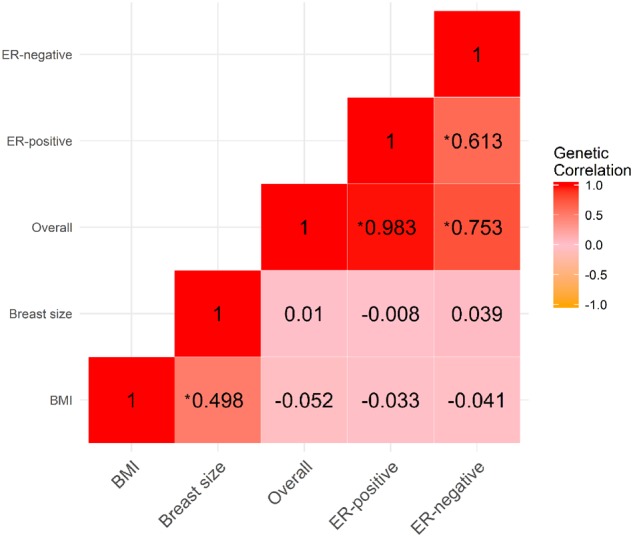
Genetic correlations between body mass index (BMI), breast size and breast cancer risk (overall, ER-positive and ER-negative). Colour intensity indicates correlation strength, with red indicating positive correlation (from 0 to +1) and yellow indicating negative correlation (from 0 to −1). Significant relationships (*P* < 0.05) are denoted by asterisks.

Genetically predicted BMI and genetically predicted breast size were correlated [correlation coefficient (*rg*) = 0.50, *P* = 3.89x10^−43^] (Figure 2 and Supplementary Table 5, available as Supplementary data at *IJE* online) but none of the other pairs of genetically predicted variables (BMI and breast cancer risk, breast size and breast cancer risk) was found by LDSC to be correlated (*P* > 0.050). As expected, the genetic correlations between the three types of breast cancer (overall, ER-positive, ER-negative) were high (*rg *>* *0.60, all *P* < 10^–85^) (Figure 2 and Supplementary Table 5, available as Supplementary data at *IJE* online).

### BMI has a positive effect on breast size but not vice versa

**Figure 3.**
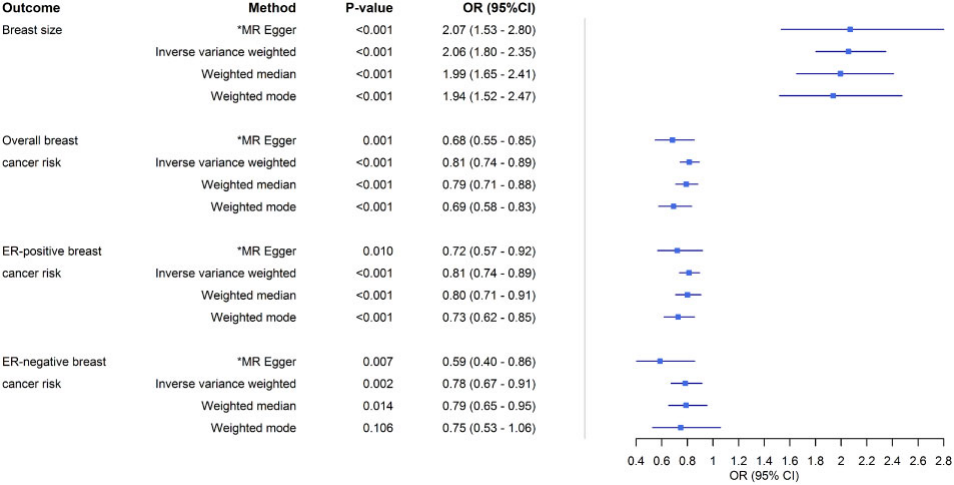
Odds ratios (ORs) and 95% confidence intervals (CI) for the association between the exposure body mass index (BMI) and two outcomes (breast size and breast cancer risk) based on the different Mendelian randomization approaches used in this study. *Value based on causal effect estimate from MR-Egger regression; corresponding MR-Egger intercept value testing presence of directional (bias inducing) pleiotropy not shown.

**Figure 4.**
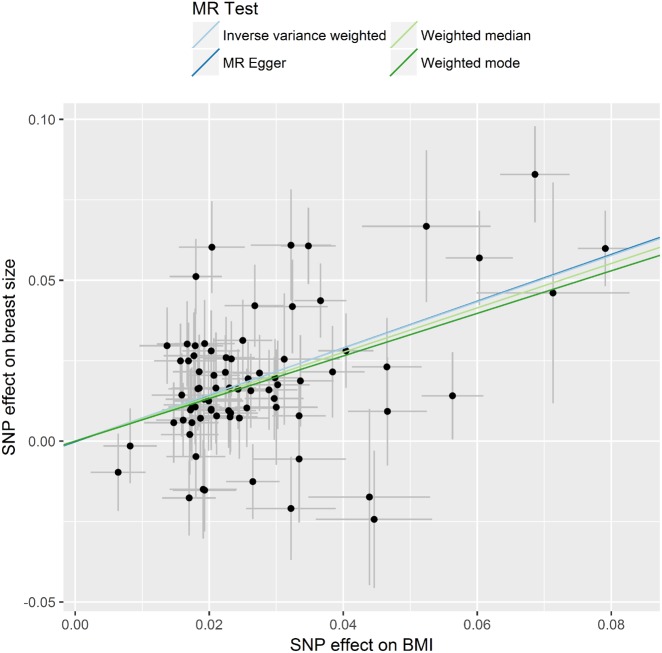
Scatter plot of SNP-breast size associations against SNP-body mass index (BMI) associations with estimates from different Mendelian randomization methods indicated by corresponding coloured lines.

**Figure 5.**
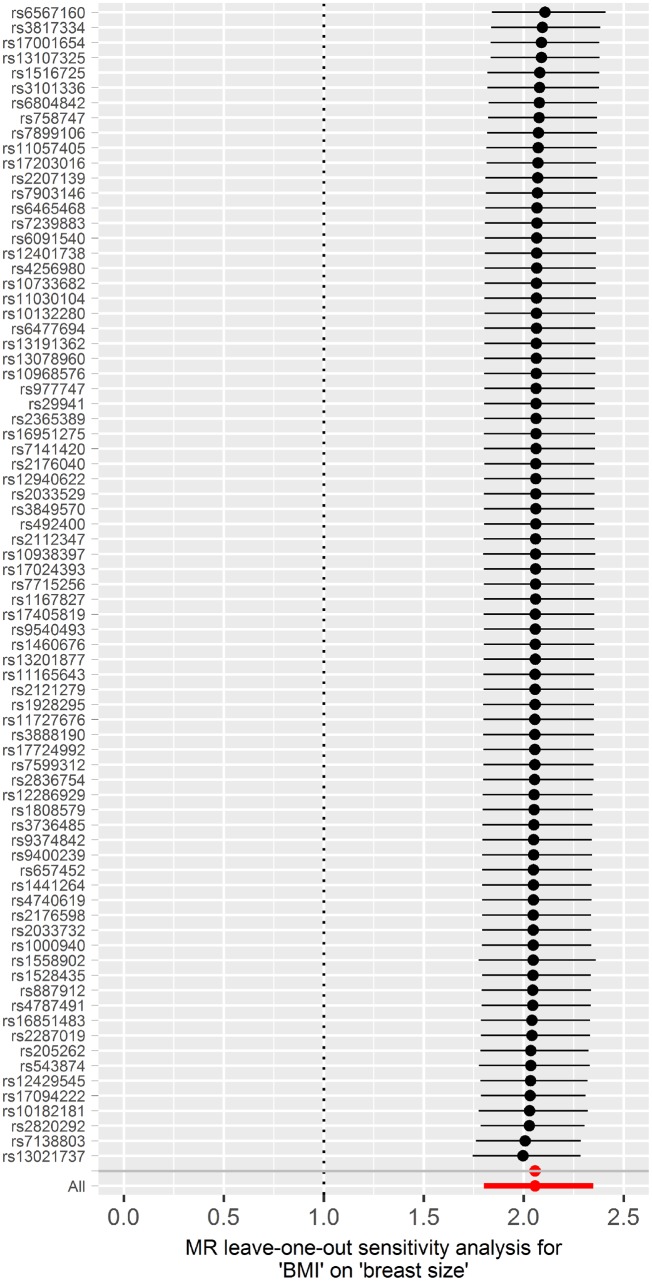
Leave-one-out permutation analysis plot for breast size obtained by leaving out the SNP indicated and repeating the standard inverse-variance weighted method with the rest of the 76 SNP instrumental variables.

A 1-SD increase in genetically predicted BMI was strongly associated with an increase in genetically predicted breast size {odds ratio (OR_IVW_) [95% confidence interval (CI)]: 2.06 (1.80 − 2.35), *P* = 1.38x10^−26^, Figure 3}. This result was corroborated by other MR methods, namely, weighted median [OR_weighted median_: 1.99 (1.64 − 2.42), *P* = 3.29x10^−12^], weighted mode [OR_weighted mode_: 1.94 (1.49–2.51), *P* = 3.82x10^−6^] and MR-Egger [OR_MR-Egger_: 2.07 (1.53 − 2.80), *P* = 1.04x10^−5^] (Figures 3 and 4). The MR-Egger intercept test (*P* = 0.964) suggested an absence of strong directional pleiotropy and leave-one-out permutation analysis (Figure 5) did not detect any single SNP that had a strong influence on the results. For the reverse direction, regression coefficients close to zero with non-significant *P*-values were obtained when breast size was used as exposure (Supplementary Figures 1 and 2, available as Supplementary data at *IJE* online).

### BMI has a negative effect on breast cancer risk but not vice versa

**Figure 6.**
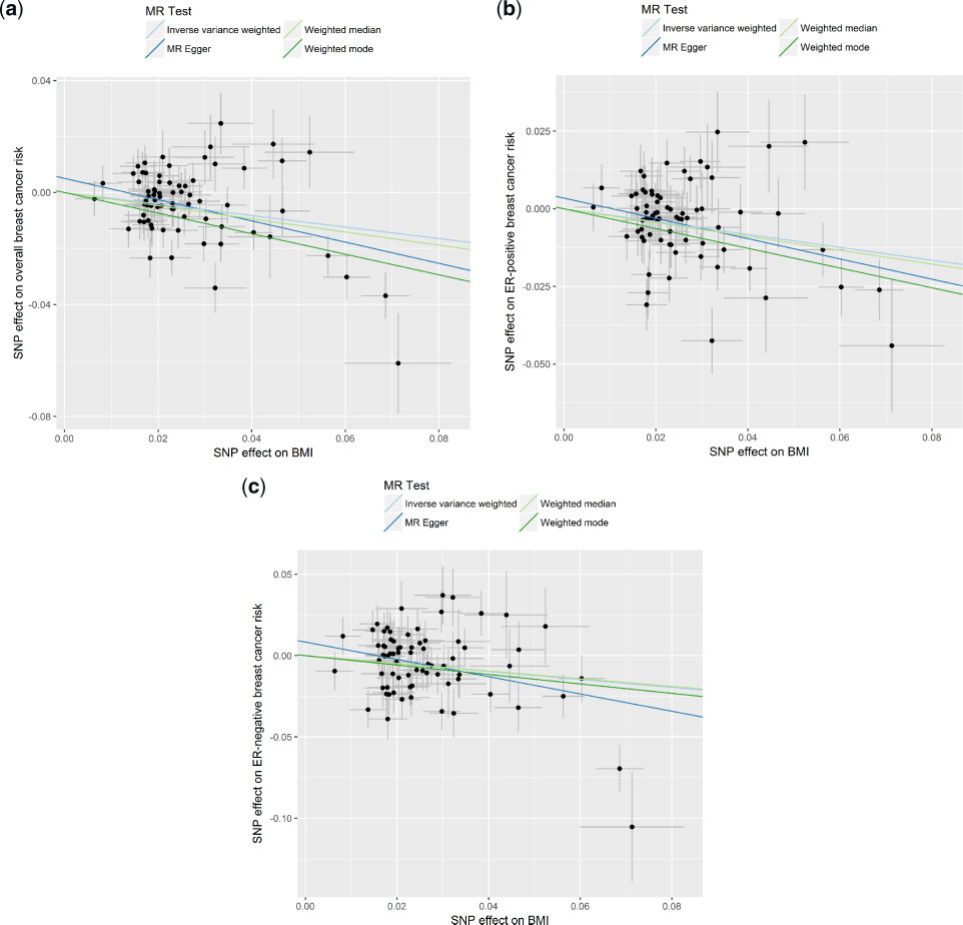
Scatter plots of SNP-breast cancer risk associations against SNP-body mass index (BMI) associations for (a) overall breast cancer risk, (b) oestrogen receptor (ER)-positive breast cancer risk, and (c) ER-negative breast cancer risk with estimates from different Mendelian randomization methods indicated by corresponding coloured lines.

**Figure 7.**
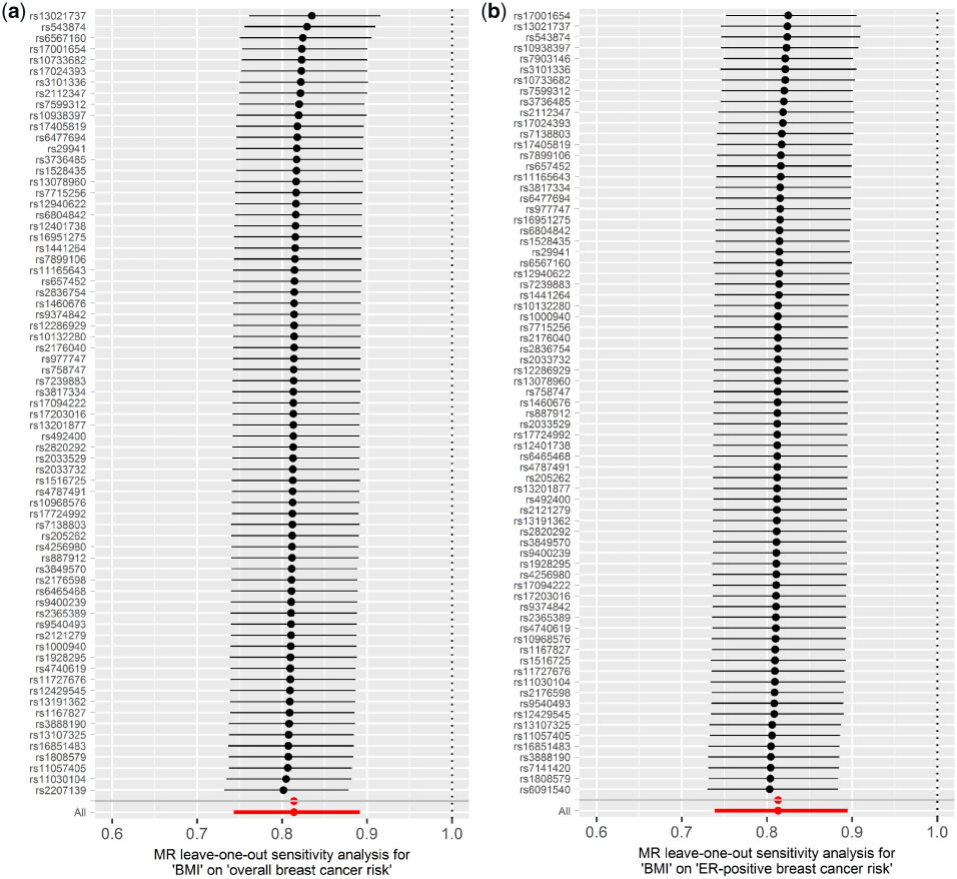

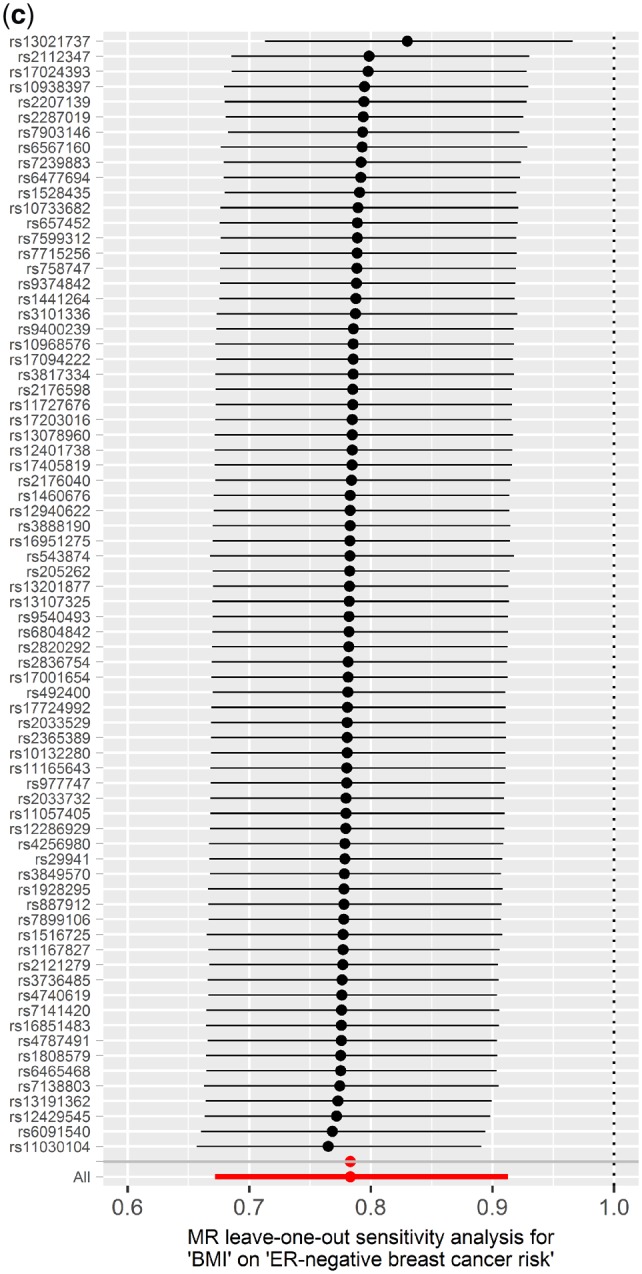
Leave-one-out permutation analysis plots for (a) overall breast cancer risk, (b) oestrogen receptor (ER)-positive breast cancer risk, and (c) ER-negative breast cancer risk obtained by leaving out the SNP indicated and repeating the standard inverse-variance weighted method with the rest of the 70, 72 and 73 SNP instrumental variables used respectively.

In the IVW analysis, an increase in genetically predicted BMI was strongly associated with a decrease in overall [OR_IVW_: 0.81 (0.74 − 0.89), *P* = 9.44x10^−6^], ER-positive [OR_IVW_: 0.79 (0.72–0.88), *P* = 4.84x10^−6^] and ER-negative [OR_IVW_: 0.78 (0.67 − 0.91), *P* = 1.78x10^−3^] breast cancer risk (Figure 3). Similarly, the estimates derived from other MR methods did not appreciably differ from the IVW method (Figures 3 and 6a–c). No evidence for directional horizontal pleiotropy was observed (*P* = 0.095, 0.219 and 0.108 for BMI-overall, BMI-ER-positive and BMI-ER-negative breast cancer risk, respectively) and none of the SNPs had an undue effect on the results (Figure 7a–c). These results were obtained using 71 SNPs (BMI-overall risk), 73 SNPs (BMI-ER-positive risk) and 74 SNPs (BMI-ER-negative risk) after removal of outliers as identified by MR-PRESSO. Before removal of outliers, the MR-Egger intercepts differed significantly from zero (*P* = 0.006, 0.026 and 0.002, respectively). For the reverse direction, regression coefficients close to zero were obtained when all three risks were used as exposure, and all *P*-values were not significant (all *P* > 0.070) (Supplementary Figures 3b, 4b, 5b and 6A–C, available as Supplementary data at *IJE* online). One of the SNPs (rs17817449) appeared to have a stronger effect compared with the other SNPs from the leave-one-out analysis (Supplementary Figure 6D–F, available as Supplementary data at *IJE* online), and this same outlier was also detected through MR-PRESSO, but removing it had a negligible effect on the results.

### Unclear relationship between breast size and breast cancer risk

**Figure 8.**
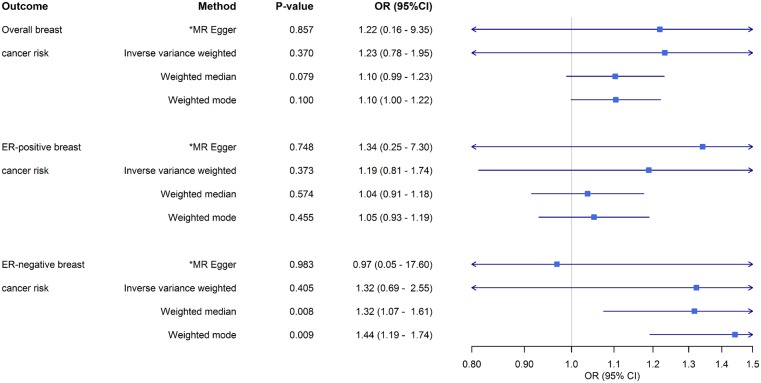
Odds ratios (ORs) and 95% confidence intervals (CI) for the association between breast size and overall breast cancer risk, ER-positive breast cancer risk, and ER-negative breast cancer risk based on the different Mendelian randomization approaches used in this study. *Value based on causal effect estimate from MR-Egger regression; corresponding MR-Egger intercept value testing presence of directional (bias inducing) pleiotropy not shown.

**Figure 9.**
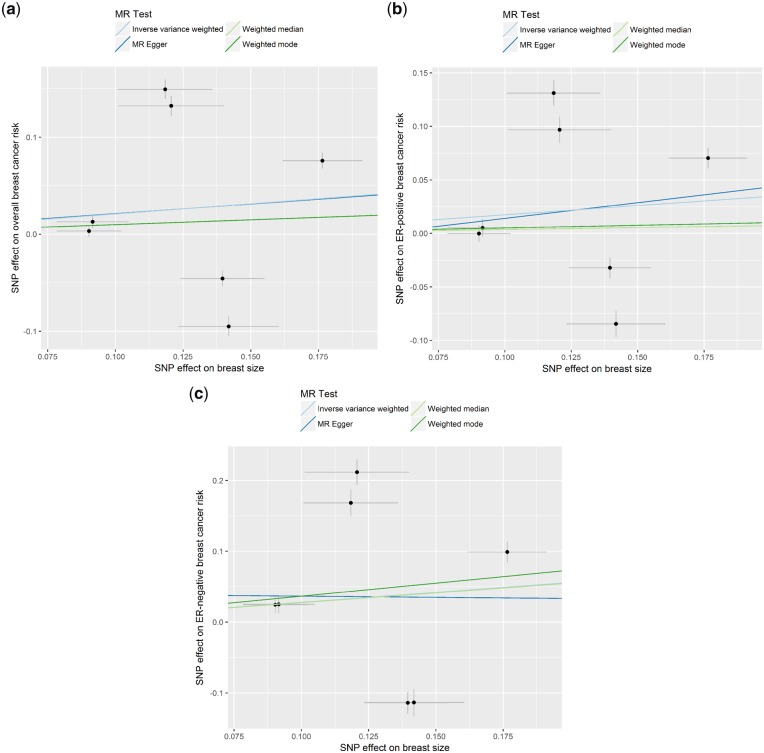
Scatter plots of SNP-breast cancer risk associations against SNP-breast size associations for (a) overall breast cancer risk, (b) oestrogen receptor (ER)-positive breast cancer risk, and (c) ER-negative breast cancer risk with estimates from different Mendelian randomization methods indicated by corresponding coloured lines.

**Figure 10.**
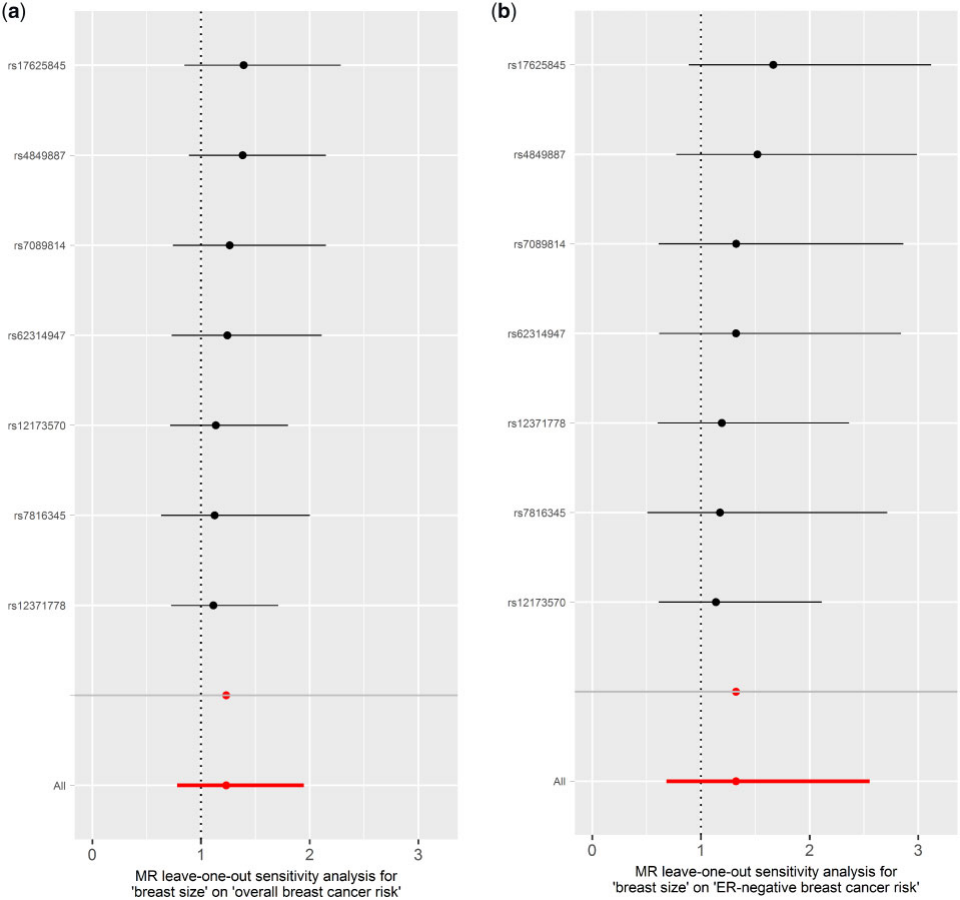

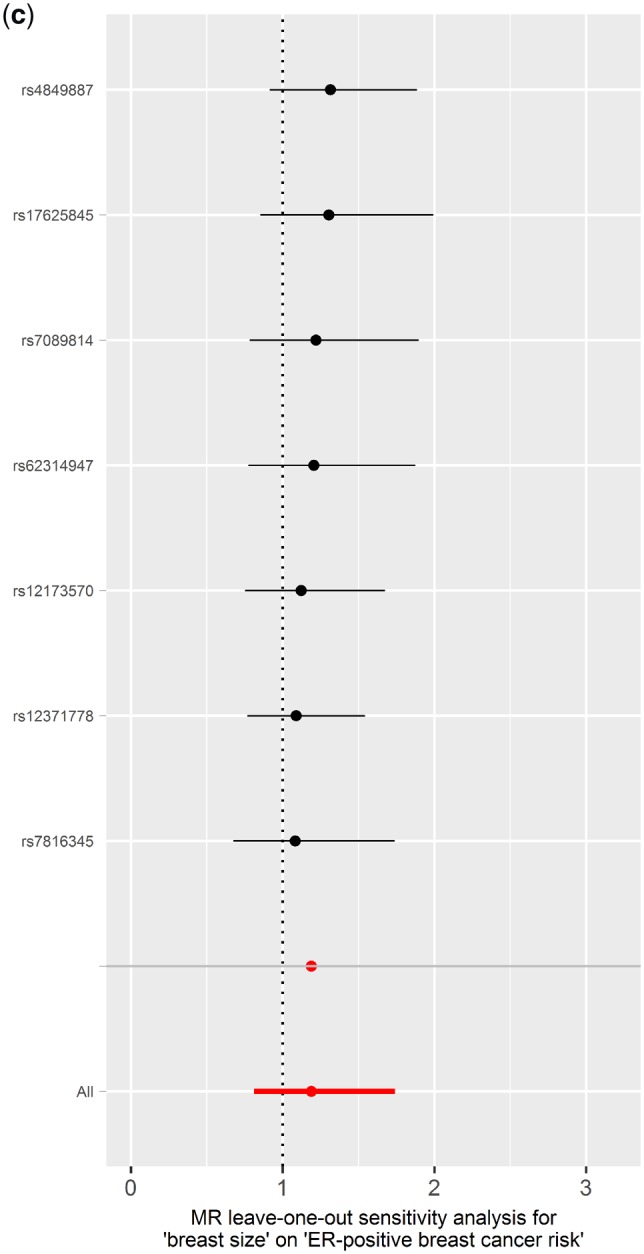
Leave-one-out permutation analysis plots for (a) overall breast cancer risk, (b) oestrogen receptor (ER)-positive breast cancer risk, and (c) ER-negative breast cancer risk obtained by leaving out the SNP indicated and repeating the standard inverse-variance weighted method with the six SNP instrumental variables used respectively.

An increase in genetically predicted breast size was not significantly associated with breast cancer risk [OR_IVW_: 1.23 (0.78−1.95), *P* = 0.370, 1.19 (0.81–1.74), *P* = 0.373 and 1.32 (0.69−2.55), *P* = 0.405 for overall, ER-positive and ER-negative breast cancer risk, respectively] (Figure 8). The OR estimates obtained from other methods were also not significant except for those obtained from the weighted median and weighted mode methods for the breast size-ER-negative association [OR_weighted median_: 1.32 (1.07−1.61), *P* = 0.008 and OR_weighted mode_: 1.44 (1.19−1.75), *P* = 0.009] (Figures 8 and 9a–c). No directional pleiotropic effect was detected as MR-Egger intercept tests did not differ significantly from zero (*P* = 0.991, 0.891 and 0.836 for size-overall, size-ER-positive and size-ER-negative breast cancer risk, respectively), and no outliers were detected by leave-one-out permutation analysis (Figure 10a–c). For the reverse direction, regression coefficients close to zero were obtained when all three breast cancer risks were used as exposure and all *P*-values were not significant (all *P* > 0.470) (Supplementary Figures 3a, 4a, 5a and 7, available as Supplementary data at *IJE* online).

One SNP (rs7816345) was reported to be associated with both BMI and breast density, which may undermine the validity of the instrument for breast size.^6^^,^^33^ In a sensitivity analysis, removing SNP rs7816345 from the MR analysis resulted in attenuated results. In other words, the effect of breast size on ER-negative breast cancer risk was no longer significant [OR_weighted median_: 1.16 (0.95–1.43), and OR_weighted mode_: 0.94 (0.73–1.23)].

## Discussion

**Figure 11.**
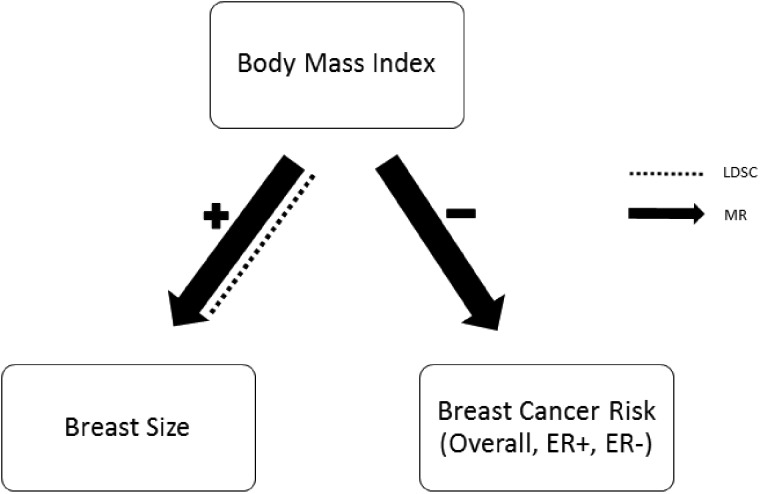
Relationships discovered in this study. Significant results from LDSC regression are denoted by dotted lines and significant results from MR analysis are denoted by solid arrows.

In this study, we found potential causal genetic evidence linking BMI to both breast size and breast cancer risk (Figure 11). Our finding that genetically predicted BMI is positively correlated with genetically predicted breast size is not surprising given that a number of other epidemiological studies have reported similar associations. Our analyses extend the finding of Wade *et al.,*^10^ who used data from twin studies to infer that one third of genes contributing to breast size were in common with genes influencing BMI. Here, we used directly all SNPs in common between the two traits to calculate their genetic correlation after adjusting for genetic linkage, and found this correlation to be large and statistically significant. To our knowledge, this is the first time that empirical genetic data have been used directly to show a positive association between BMI and breast size. Additionally, our MR results suggest that a genetic predisposition to higher BMI may be causally linked to larger breast size, but not the reverse. This direction of association implies that women with higher BMI have larger breast size.

We also found through MR analyses a significant relationship between genetically predicted BMI and breast cancer risk. Higher genetically predicted BMI was found to be inversely associated with the risk of developing both ER-positive and ER-negative breast cancers. Some studies have suggested that BMI is more strongly associated with hormone receptor-positive cancers,^14^^,^^15^ but we did not observe this. Furthermore, although ER-negative breast cancers are more common in premenopausal women, we did not find any differences in the (negative) association between BMI and breast cancer by ER-status. This contradicts the findings from other observational studies that suggest that BMI is positively associated with postmenopausal breast cancer but negatively associated with premenopausal breast cancer.^11–13^ It is important to note that the GWAS summary statistics we used did not directly differentiate between pre- and post-menopausal breast cancer cases. Nevertheless, our findings are consistent with the MR analyses of Shu *et al*.^19^ Gao *et al.*^18^ and Guo *et al.*^17^ who, using genetic data, found that BMI was inversely associated with both pre- and post-menopausal breast cancer risk. Given that other studies using a similar methodology have reported this inverse association and that it was independent of age, we were not overly surprised by the results. The protective effect obtained in our study (OR = 0.81) was weaker than that found by Guo *et al.* (OR = 0.65) but this difference could be due to the different datasets used as well as Guo *et al.*’s use of a genetic risk score as an instrumental variable instead of the beta-coefficients of each SNP that we used in our study.

The positive association between BMI and breast cancer risk in postmenopausal women has been speculated to result from the higher tissue concentrations of oestrogen derived from the larger fat reserves of women with higher BMI.^34^ On the other hand, the negative relationship between BMI and breast cancer risk in premenopausal women might be explained by lower levels of progesterone and oestrogen due to the longer anovulatory cycles that they experience.^35^^,^^36^ In explaining why genetically predicted BMI was inversely correlated with postmenopausal risk, Guo *et al.*^17^ suggest that the genetic portion of BMI may reflect an early-life BMI, or that weight gain during later adulthood and not BMI per se, could be the main factor leading to increased postmenopausal risk. These reasons could also explain the results obtained from our MR analyses. Interestingly, whereas a negative correlation was estimated between BMI and breast cancer risk using LDSC, it was not significant (Supplementary Table 5, available as Supplementary data at *IJE* online). Also, MR analysis using breast cancer risk as the exposure and BMI as the outcome did not produce any significant associations. Thus, the association between BMI and breast cancer risk is mediated by only a limited number of SNPs that are significant for BMI but not for breast cancer risk.

Breast size is not a commonly considered breast cancer risk factor.^1^ Indeed, the relationship between our final pair of traits, breast size and breast cancer risk, is less clear. Eriksson *et al.*^6^ reported two SNPs associated with breast size that were also associated with breast cancer risk, but when we used all seven SNPs for MR analysis, we were unable to detect a significant correlation except when using the weighted median and weighted mode methods. For these two methods, there was a positive relationship only between breast size and ER-negative breast cancer risk. The weighted median and weighted mode methods work on the assumption that some of the instrumental variables do not satisfy the requirements for valid MR analysis, but given that only seven SNPs were used for the analysis, this result has to be interpreted with caution. In addition, the average pleiotropy was not significantly different from 0, which may imply that there is no directional pleiotropy and that the estimate from the IVW method is not biased (i.e. results were consistent with the weighted median and weighted mode methods). Furthermore, when we used all SNPs for LDSC regression, no relationship was found as well. Taken together, there is no clear genetic evidence for a relationship between breast size and breast cancer risk.

Indeed, the relationship between breast size and breast cancer risk may be difficult to tease apart without the consideration of mammographic density, which is a strong, independent risk factor of breast cancer.^37^ Total breast size (i.e. area) on a mammogram is a combination of radiologically dense and non-dense components. Absolute non-dense area on a mammogram is highly correlated with total breast area and is inversely associated with breast cancer risk.^38^ Radiologically dense tissue, on the other hand, contributes to breast cancer risk.^38^ It is unclear whether the breast size instrument currently used is catching size, density or a mixture of both. Future MR analyses examining relationships between mammographic density measures (i.e. percentage mammographic density, absolute dense area and absolute non-dense area) and breast cancer risk will be meaningful.

It is important to note other limitations to our analysis. Firstly, due to the use of summary statistics, we were not able to stratify our analyses based on BMI or other breast cancer risk factors. This could have provided further insight into the nature of the relationship between these two traits. Secondly, the association between breast size and breast cancer risk could be driven by environmental factors that have no genetic component making it undetectable in this study. Thirdly, the seven SNPs were identified using a semi-continuous measurement of bra cup size to measure breast size.^6^ The lower variability in bra cup size as compared with a continuous variable, such as breast area, may have resulted in fewer SNPs being identified. However, the amount of variation in a phenotypic trait that is explained by genetic variants used in MR analyses is typically small (<1%, compared with ∼1.2% for breast size).^39^

Our study is the first to address the potentially causal relationship between breast size and breast cancer using a MR approach. In summary, our results suggest that women with a genetic predisposition to high BMI are likely to have a larger breast size and a lower risk of breast cancer, but there is no clear evidence for a direct and unmediated association between breast size and breast cancer risk. Interestingly, the reverse causation of breast cancer risk to BMI, breast cancer risk to breast size and breast size to BMI were all not significant, which is in agreement with our intuition. The hypothesis that breast cancer risk is lower in women with both a larger breast size as well as a genetic predisposition for high BMI can be tested in future stratified studies.

## Funding

This work was supported by the National Research Foundation Singapore Fellowship (NRF-NRFF2017-02) awarded to J.L. D.G.E is a NIHR Senior investigator. The body mass index association data were obtained from the Genetic Investigation of ANthropometric Traits (GIANT) consortium, whereas the breast cancer genome-wide association meta-analyses, which generated the summary statistics used here, were supported by the Government of Canada through Genome Canada and the Canadian Institutes of Health Research, the ‘Ministère de l’Économie, de la Science et de l’Innovation du Québec’ through Genome Québec and grant PSR-SIIRI-701, The US National Institutes of Health (U19 CA148065, X01HG007492), Cancer Research UK (C1287/A10118, C1287/A16563, C1287/A10710) and The European Union (HEALTH-F2-2009–223175 and H2020 633784 and 634935). All studies and funders for the breast cancer genome-wide association analyses are listed in Michailidou *et al*.^24^

## Supplementary Material

dyz124_Supplementary_Material
