# Supplementary material for: The genetic interplay between body mass index, breast size and breast cancer risk: a Mendelian randomization analysis
Source: Int J Epidemiol. 2019 Jun 26;48(3):781–94. doi: 10.1093/ije/dyz124 (PMC6659372; doi:10.1093/ije/dyz124)
Supplement: dyz124_Supplementary_Material [file dyz124_supplementary_material.pdf]

The genetic interplay between body mass index, breast size and breast cancer risk: A Mendelian randomization analysis

Supplementary Material

#Brandon Nick Sern Ooi[1] brandon_ooi@gis.a-star.edu.sg

#Huiwen Loh[1] loh_hui_wen_from.tp@gis.a-star.edu.sg

Peh Joo Ho[1] ho_peh_joo@gis.a-star.edu.sg

Roger Milne[2] Roger.Milne@cancervic.org.au

Graham Giles[2] Graham.Giles@cancervic.org.au

Chi Gao[3] chg745@mail.harvard.edu

Peter Kraft[3] pkraft@hsph.harvard.edu

Esther M. John[4] emjohn@stanford.edu

Anthony Swerdlow[5] Anthony.Swerdlow@icr.ac.uk

Hermann Brenner[6] h.brenner@Dkfz-Heidelberg.de

Anna H. Wu[7] Anna.Wu@med.usc.edu

Christopher Haiman[7] Christopher.Haiman@med.usc.edu

D. Gareth Evans[8] Gareth.Evans@mft.nhs.uk

Wei Zheng[9] wei.zheng@Vanderbilt.Edu

Peter Fasching[10] Peter.Fasching@uk-erlangen.de

Jose Esteban Castelao[11] Esteban.Castelao@med.usc.edu

Ava Kwong[12] avakwong@hku.hk

Shen Xia[13] xia.shen@ki.se

Kamila Czene[13] Kamila.Czene@ki.se

Per Hall[13] Per.Hall@ki.se

Alison Dunning[14] amd24@medschl.cam.ac.uk

Douglas Easton[14] dfe20@medschl.cam.ac.uk

Mikael Hartman[15] ephbamh@nus.edu.sg

*Jingmei Li[1,15] lijm1@gis.a-star.edu.sg

#These authors contributed equally

[1] Genome Institute of Singapore, 60 Biopolis Street, Genome, #02-01, Singapore 138672, Singapore

[2] Cancer Epidemiology & Intelligence Division, Cancer Council Victoria, Melbourne, VIC, Australia

[3] Program in Genetic Epidemiology and Statistical Genetics, Harvard T.H. Chan School of Public Health

[4] Department of Medicine and Stanford Cancer Institute, Stanford University School of Medicine, Stanford, CA, USA

[5] The Institute of Cancer Research, University of London

[6] Clinical Epidemiology and Aging Research, German Cancer Research Center (DKFZ)

[7] Department of Preventive Medicine, Keck School of Medicine, University of Southern California, Los Angeles, CA, USA

[8] Division of Evolution & Genomic Sciences, The University of Manchester

[9] Vanderbilt Epidemiology Center, Vanderbilt University Medical Centre, Vanderbilt University

[10] Friedrich–Alexander University Erlangen–Nürnberg

[11]Oncology and Genetics Unit, Instituto de Investigacion Sanitaria Galicia Sur (IISGS), Xerencia de Xestion Integrada de Vigo-SERGAS, Vigo 36312, Spain

[12] Department of Surgery, The University of Hong Kong

[13] Department of Medical Epidemiology and Biostatistics, Karolinska Insititute

[14] Centre for Cancer Genetic Epidemiology, University of Cambridge

[15] Department of Surgery, Yong Loo Lin School of Medicine, National University of Singapore

*__Correspondence to:__ Jingmei Li, PhD, Genome Institute of Singapore, 60 Biopolis Street, Genome, #02-01, Singapore 138672, Singapore. Tel: (65) 6808 8312; Email: lijm1@gis.a-star.edu.sg

Supplementary Table 1. Description of each genome-wide association (GWAS) dataset used in the estimation of genetic correlations. ER: estrogen receptor.

Trait	File	Unit of measurement	Controls	Cases	N	Total SNP from downloaded summary statistics file	SNPs merge with HapMap3 (excluding duplicated SNPs)	GWAS-sig SNPs
Breast cancer risk								
Overall	Breast Cancer Association Consortium (BCAC): oncoarray_bcac_public_release_oct17.txt.gz	Coded as binary phenotype	105,974	122,977	228,951	10,783,221	1,139,935	2,832
ER-positive	Breast Cancer Association Consortium (BCAC): oncoarray_bcac_public_release_oct17.txt.gz	Coded as binary phenotype	105,974	69,501	175,475	10,783,221	1,139,934	2,217
ER-negative	Breast Cancer Association Consortium (BCAC): oncoarray_bcac_public_release_oct17.txt.gz	Coded as binary phenotype	105,974	21,468	127,442	10,783,221	1,139,934	350
Breast size	23andMe (version 4.1)	Per unit of bra cup size			33,790	13,794,599	1,208,378	216
BMI	GIANT 2015 women only summary statistics file: Women_SNP_gwas_mc_merge_nogc.tbl.uniq.gz	kg per m^2			183,507	2,479,430	1,063,724	523

Supplementary Table 2. 77 body mass index variants (SNP) and corresponding effect allele, frequency (EAF), effect size (Beta) and standard error (SE). Chr: chromosome. The first four columns indicate whether the SNP was present in other datasets after processing and harmonization.

	Breast cancer risk											
**Breast size**	**Overall**	**ER-positive**	**ER-negative**	**SNP**	**Chr**	**Gene**	**Effect Allele**	**Other Allele**	**EAF**	**Beta**	**SE**	**P-Value**
Yes	Yes	Yes	Yes	rs11165643	1	PTBP2	C	T	0.425	-0.023	0.004	5.76E-09
Yes	Yes	Yes	Yes	rs12401738	1	FUBP1	A	G	0.425	0.0256	0.0041	6.09E-10
Yes	Yes	Yes	Yes	rs17024393	1	GNAT2	C	T	0.04167	0.0713	0.0114	3.53E-10
Yes	Yes	Yes	Yes	rs2820292	1	NAV1	A	C	0.4917	-0.018	0.0039	4.89E-06
Yes	Yes	Yes	Yes	rs3101336	1	NEGR1	T	C	0.3509	-0.0334	0.004	8.91E-17
Yes	Yes	Yes	Yes	rs543874	1	SEC16B	G	A	0.2667	0.0603	0.005	9.61E-34
Yes	Yes	Yes	Yes	rs657452	1	AGBL4	A	G	0.4167	0.0233	0.0041	1.70E-08
Yes	Yes	Yes	Yes	rs977747	1	TAL1	T	G	0.4667	0.0174	0.004	1.11E-05
Yes		Yes		rs10182181	2	ADCY3	A	G	0.5	-0.0366	0.0039	3.91E-21
Yes	Yes	Yes	Yes	rs13021737	2	TMEM18	A	G	0.125	-0.0686	0.0052	6.99E-40
Yes	Yes	Yes	Yes	rs1460676	2	FIGN	T	C	0.7833	-0.0199	0.0052	0.00012
Yes	Yes	Yes	Yes	rs1528435	2	UBE2E3	T	C	0.5833	0.0137	0.0041	0.00084
Yes	Yes	Yes	Yes	rs17203016	2	CREB1	G	A	0.2	0.0191	0.005	0.000149
Yes	Yes	Yes	Yes	rs2121279	2	LRP1B	T	C	0.1167	0.021	0.0056	0.000174
Yes	Yes	Yes	Yes	rs2176040	2	LOC646736	G	A	0.6083	-0.0064	0.0041	0.1191
Yes	Yes	Yes	Yes	rs492400	2	USP37	T	C	0.675	-0.0082	0.004	0.03814
Yes	Yes	Yes	Yes	rs7599312	2	ERBB4	G	A	0.7083	0.0183	0.0044	2.69E-05
Yes	Yes	Yes	Yes	rs887912	2	Intergenic	T	C	0.3167	0.025	0.0044	1.49E-08
Yes	Yes	Yes	Yes	rs13078960	3	CADM2	T	G	0.8167	-0.0336	0.005	1.41E-11
Yes	Yes	Yes	Yes	rs1516725	3	ETV5	T	C	0.0917	-0.0466	0.0059	1.91E-15
Yes	Yes	Yes	Yes	rs16851483	3	RASA2	G	T	0.9083	-0.0524	0.0096	4.81E-08
Yes	Yes	Yes	Yes	rs2365389	3	FHIT	C	T	0.6583	0.0203	0.004	2.98E-07
Yes	Yes	Yes	Yes	rs3849570	3	GBE1	A	C	0.3667	0.0147	0.0044	0.000947
Yes	Yes	Yes	Yes	rs6804842	3	RARB	A	G	0.425	-0.017	0.004	1.63E-05
Yes	Yes	Yes	Yes	rs10938397	4	GNPDA2	A	G	0.5667	-0.0404	0.0041	2.98E-23
Yes	Yes	Yes	Yes	rs11727676	4	HHIP	C	T	0.075	-0.0312	0.0083	0.000182
Yes	Yes	Yes	Yes	rs13107325	4	SLC39A8	C	T	0.8833	-0.0446	0.0087	2.90E-07
Yes	Yes	Yes	Yes	rs17001654	4	SCARB2	C	G	0.8417	-0.0322	0.0067	1.54E-06
Yes	Yes	Yes	Yes	rs2112347	5	POC5	G	T	0.375	-0.0298	0.0041	3.15E-13
Yes	Yes	Yes	Yes	rs7715256	5	GALNT10	G	T	0.45	0.0179	0.004	5.82E-06

Yes	Yes	Yes	Yes	rs13191362	6	PARK2	A	G	0.8	0.03	0.0062	1.15E-06
Yes	Yes	Yes	Yes	rs13201877	6	IFNGR1	A	G	0.9167	-0.0192	0.0057	0.000808
Yes	Yes	Yes	Yes	rs2033529	6	TDRG1	G	A	0.2583	0.0203	0.0043	2.83E-06
Yes	Yes	Yes	Yes	rs205262	6	C6orf106	A	G	0.7333	-0.0268	0.0045	2.04E-09
Yes	Yes		Yes	rs2207139	6	TFAP2B	G	A	0.1	0.0465	0.0052	2.40E-19
Yes	Yes	Yes	Yes	rs9374842	6	LOC285762	T	C	0.7417	0.0177	0.0045	8.54E-05
Yes	Yes	Yes	Yes	rs9400239	6	FOXO3	C	T	0.7	0.0225	0.0043	1.29E-07
Yes	Yes	Yes	Yes	rs1167827	7	HIP1	A	G	0.4583	-0.0172	0.0042	3.69E-05
Yes	Yes	Yes	Yes	rs6465468	7	ASB4	G	T	0.675	-0.0245	0.0045	4.98E-08
Yes	Yes	Yes	Yes	rs17405819	8	HNF4G	C	T	0.3667	-0.0243	0.0043	1.45E-08
Yes	Yes	Yes	Yes	rs2033732	8	RALYL	C	T	0.7583	0.0193	0.0045	2.08E-05
Yes	Yes	Yes	Yes	rs10733682	9	LMX1B	A	G	0.425	0.0229	0.0041	1.67E-08
Yes	Yes	Yes	Yes	rs10968576	9	LINGO2	G	A	0.2917	0.0289	0.0043	1.04E-11
Yes	Yes	Yes	Yes	rs1928295	9	TLR4	C	T	0.425	-0.0258	0.0039	3.42E-11
Yes	Yes	Yes	Yes	rs4740619	9	CCDC171	T	C	0.5333	0.0157	0.0039	5.22E-05
Yes	Yes	Yes	Yes	rs6477694	9	EPB41L4B	C	T	0.3583	0.0211	0.0041	2.77E-07
Yes	Yes	Yes	Yes	rs17094222	10	HIF1AN	C	T	0.2083	0.0204	0.0049	3.22E-05
Yes	Yes	Yes	Yes	rs7899106	10	GRID1	A	G	0.95	-0.0439	0.0091	1.60E-06
Yes		Yes	Yes	rs7903146	10	TCF7L2	T	C	0.25	-0.018	0.0044	3.97E-05
Yes	Yes	Yes	Yes	rs11030104	11	BDNF	A	G	0.8	0.0384	0.0048	2.32E-15
Yes	Yes	Yes	Yes	rs12286929	11	CADM1	G	A	0.4333	0.0207	0.0039	8.95E-08
Yes	Yes	Yes	Yes	rs2176598	11	HSD17B12	T	C	0.2	0.0167	0.0045	0.00021
Yes	Yes	Yes	Yes	rs3817334	11	MTCH2	C	T	0.55	-0.0265	0.004	2.47E-11
Yes	Yes	Yes	Yes	rs4256980	11	TRIM66	G	C	0.725	0.0232	0.0041	1.65E-08
Yes	Yes	Yes	Yes	rs11057405	12	CLIP1	A	G	0.0917	-0.0334	0.007	1.66E-06
Yes	Yes	Yes	Yes	rs7138803	12	BCDIN3D	G	A	0.5583	-0.0348	0.0041	1.80E-17
Yes	Yes	Yes	Yes	rs12429545	13	OLFM4	G	A	0.9	-0.0322	0.006	9.47E-08
Yes	Yes	Yes	Yes	rs1441264	13	MIR548A2	A	G	0.55	0.0169	0.0041	4.37E-05
Yes	Yes	Yes	Yes	rs9540493	13	MIR548X2	G	A	0.55	-0.0174	0.0042	3.01E-05
Yes	Yes	Yes	Yes	rs10132280	14	STXBP6	A	C	0.3333	-0.0171	0.0043	8.10E-05
Yes		Yes	Yes	rs7141420	14	NRXN3	T	C	0.6167	0.0262	0.0039	1.45E-11
Yes	Yes	Yes	Yes	rs16951275	15	MAP2K5	C	T	0.225	-0.0302	0.0048	2.96E-10
Yes	Yes	Yes	Yes	rs3736485	15	DMXL2	A	G	0.425	0.0185	0.0039	2.68E-06
Yes				rs1558902	16	FTO	A	T	0.45	0.0791	0.0041	4.03E-84
Yes	Yes	Yes	Yes	rs3888190	16	ATP2A1	A	C	0.3583	0.0275	0.004	3.51E-12

Yes	Yes	Yes	Yes	rs4787491	16	INO80E	A	G	0.386	-0.0179	0.0042	2.23E-05
Yes	Yes	Yes	Yes	rs758747	16	NLRC3	C	T	0.7333	-0.0193	0.0047	3.57E-05
Yes	Yes	Yes		rs1000940	17	RABEP1	G	A	0.225	0.0203	0.0043	2.20E-06
Yes	Yes	Yes	Yes	rs12940622	17	RPTOR	A	G	0.4583	-0.0161	0.004	4.76E-05
Yes	Yes		Yes	rs1808579	18	C18orf8	T	C	0.475	-0.0224	0.0039	1.23E-08
Yes	Yes	Yes	Yes	rs6567160	18	MC4R	C	T	0.2833	0.0563	0.0046	5.08E-34
Yes	Yes	Yes	Yes	rs7239883	18	LOC284260	G	A	0.3167	0.0231	0.0041	1.51E-08
Yes	Yes	Yes	Yes	rs17724992	19	PGPEP1	A	G	0.6917	0.0159	0.0045	0.000482
Yes			Yes	rs2287019	19	QPCTL	C	T	0.85	0.0324	0.0053	8.60E-10
Yes	Yes	Yes	Yes	rs29941	19	KCTD15	A	G	0.3333	-0.0187	0.0042	7.75E-06
Yes		Yes	Yes	rs6091540	20	ZFP64	C	T	0.725	0.0297	0.0044	2.15E-11
Yes	Yes	Yes	Yes	rs2836754	21	ETS2	C	T	0.65	0.0185	0.0041	6.83E-06

Supplementary Table 3. 7 breast size variants (SNP) and corresponding effect allele, frequency (EAF), effect size (Beta) and standard error (SE). Chr: chromosome. The first two columns indicate whether the SNP was present in other datasets after processing and harmonization.

Breast cancer risk	BMI	SNP	Chr	Gene	Effect allele	Other allele	EAF	Beta	SE	P-value
Yes	Yes	rs17625845	2	INHBB	C	T	0.205	1.40E-01	1.55E-02	2.22E-19
Yes	Yes	rs4849887	2	INHBB	T	C	0.113	1.42E-01	1.87E-02	3.02E-14
Yes		rs62314947	4	AREG	T	C	0.281	-9.16E-02	1.33E-02	5.85E-12
Yes		rs12173570	6	ESR1	T	C	0.101	1.21E-01	1.94E-02	5.52E-10
Yes	Yes	rs7816345	8	ZNF703	T	C	0.194	-1.76E-01	1.47E-02	3.31E-33
Yes	Yes	rs7089814	10	ZNF365	C	T	0.375	9.03E-02	1.19E-02	3.23E-14
Yes		rs12371778	12	PTHLH	G	C	0.091	-0.11839	1.76E-02	1.62E-11

Supplementary Table 4. 114 breast cancer risk variants (SNP) and corresponding effect allele, frequency (EAF), effect size (Beta) and standard error (SE). Chr: chromosome. The first two columns indicate whether the SNP was present in other datasets after processing and harmonization.

Breast Size	BMI	SNP	Chr	Gene	Other allele	Effect allele	Overall breast cancer risk				ER-positive breast cancer risk				ER-negative breast cancer risk			
							EAF	Beta	SE	P-Value	EAF	Beta	SE	P-Value	EAF	Beta	SE	P-Value
Yes	Yes	rs11117758	1	ESRRG	G	A	0.2086	-0.0449	0.0076	3.90E-09	0.2082	-0.05	0.0091	4.06E-08	0.209	-0.0125	0.0138	0.3654
Yes	Yes	rs11552449	1	AP4B1 / DCLRE1B	C	T	0.1658	0.0543	0.0082	4.64E-11	0.1658	0.0557	0.0098	1.23E-08	0.1658	0.0501	0.015	0.000843
Yes		rs12048493	1	OTUD7B	A	C	0.3813	0.0496	0.0067	8.62E-14	0.3815	0.0575	0.0079	3.39E-13	0.3803	0.0396	0.0123	0.001318
Yes	Yes	rs12405132	1	RNF115	C	T	0.369	-0.0406	0.0066	6.30E-10	0.3691	-0.0481	0.0077	5.60E-10	0.369	-0.0157	0.0118	0.1825
Yes	Yes	rs1707302	1	LOC101929626 /LOC110117498-PIK3R3 /PIK3R3	A	G	0.6641	0.0364	0.0066	2.95E-08	0.664	0.0466	0.0078	2.75E-09	0.6641	-0.0067	0.0119	0.5727
Yes	Yes	rs17426269	1		G	A	0.1488	0.0487	0.0086	1.74E-08	0.1488	0.0576	0.0103	1.89E-08	0.1488	0.0153	0.0158	0.3349
Yes		rs35383942	1	PHLDA3	C	T	0.0562	0.101	0.0139	3.79E-13	0.0561	0.0918	0.0164	2.33E-08	0.0564	0.0905	0.0252	0.00033
Yes		rs4233486	1		C	T	0.6514	0.0396	0.0069	9.09E-09	0.6508	0.0441	0.0082	7.93E-08	0.6511	0.0355	0.0125	0.00444
Yes	Yes	rs616488	1	PEX14	A	G	0.3313	-0.0604	0.0066	5.03E-20	0.3302	-0.0417	0.0078	1.08E-07	0.3307	-0.105	0.0121	4.12E-18
Yes	Yes	rs6678914	1	LGR6	G	A	0.4111	-0.0066	0.0063	0.295	0.4112	0.018	0.0076	0.01745	0.4106	-0.0823	0.0118	2.60E-12
Yes		rs72755295	1	EXO1	A	G	0.0309	0.1376	0.0179	1.65E-14	0.0311	0.1481	0.0208	1.08E-12	0.031	0.1183	0.0334	0.000391
Yes	Yes	rs7529522	1		T	C	0.2338	0.0478	0.0075	1.73E-10	0.234	0.0438	0.0088	6.88E-07	0.234	0.0545	0.0134	4.83E-05
Yes		rs113577745	2		C	G	0.0992	0.064	0.0102	3.91E-10	0.099	0.0622	0.0122	3.41E-07	0.099	0.05	0.0187	0.007354
Yes		rs12479355	2		A	G	0.2088	-0.0426	0.0076	2.36E-08	0.2091	-0.0424	0.0091	3.48E-06	0.2087	-0.0109	0.0139	0.4331
Yes	Yes	rs12710696	2		T	C	0.6381	-0.0365	0.0064	1.26E-08	0.6379	-0.0158	0.0076	0.03897	0.6374	-0.0628	0.0116	6.49E-08
Yes	Yes	rs16857609	2	DIRC3	C	T	0.2616	0.0727	0.007	1.82E-25	0.2621	0.0721	0.0083	3.43E-18	0.2618	0.0713	0.0127	1.83E-08
Yes		rs1830298	2	ALS2CR12	C	T	0.72	-0.0561	0.0068	1.87E-16	0.7195	-0.0519	0.0081	1.57E-10	0.7192	-0.0613	0.0124	7.40E-07
Yes		rs2016394	2	DLX2-AS1	G	A	0.4748	-0.0425	0.0062	6.23E-12	0.4753	-0.0603	0.0074	3.22E-16	0.4742	0.0084	0.0113	0.4559
Yes	Yes	rs4442975	2	LOC101928278	G	T	0.5021	-0.1274	0.0061	1.14E-95	0.5018	-0.1482	0.0073	6.65E-91	0.5004	-0.0521	0.0112	3.38E-06
Yes	Yes	rs4849887	2		T	C	0.899	0.095	0.0104	6.92E-20	0.8989	0.0845	0.0124	1.01E-11	0.899	0.1135	0.0193	4.13E-09

Yes		rs6725517	2	ADCY3	A	G	0.4088	-0.0468	0.0067	2.93E-12	0.4077	-0.0385	0.008	1.59E-06	0.4065	-0.0683	0.0122	1.98E-08
Yes	Yes	rs1053338	3	ATXN7	A	G	0.1349	0.0588	0.009	5.31E-11	0.1349	0.0554	0.0107	2.12E-07	0.1352	0.0366	0.0163	0.02457
Yes	Yes	rs12493607	3	TGFBR2	G	C	0.3444	0.0485	0.0065	6.91E-14	0.3443	0.0608	0.0077	2.92E-15	0.3448	0.0016	0.0118	0.8955
Yes		rs13066793	3	VGLL3	A	G	0.0925	-0.0685	0.0112	1.04E-09	0.0931	-0.0687	0.0133	2.52E-07	0.0921	-0.0477	0.0211	0.02346
Yes	Yes	rs4973768	3	SLC4A7	C	T	0.4713	0.0985	0.0062	4.79E-57	0.4714	0.1086	0.0074	5.11E-49	0.4725	0.0413	0.0112	0.00024
Yes		rs58058861	3	LINC02068	G	A	0.2172	0.0474	0.0074	1.91E-10	0.2172	0.0519	0.0089	4.75E-09	0.2165	-0.0025	0.0137	0.8526
Yes	Yes	rs6762644	3	ITPR1	A	G	0.3888	0.055	0.0063	4.02E-18	0.3875	0.0605	0.0076	1.17E-15	0.3871	0.0225	0.0116	0.0525
Yes		rs6796502	3		G	A	0.0986	-0.0828	0.0106	5.54E-15	0.0985	-0.0892	0.0127	2.22E-12	0.0982	-0.0663	0.0194	0.000649
Yes		rs6805189	3	FOXP1	T	C	0.4751	-0.0339	0.0062	4.60E-08	0.4749	-0.0407	0.0074	3.76E-08	0.4751	-0.0291	0.0113	0.009938
Yes	Yes	rs9833888	3	CMSS1/ FILIP1L	G	T	0.2259	0.0457	0.0074	5.15E-10	0.226	0.0542	0.0088	6.15E-10	0.2251	0.0155	0.0135	0.2487
Yes	Yes	rs10022462	4	LOC105369192	C	T	0.4379	0.0375	0.0062	1.55E-09	0.4376	0.0396	0.0074	9.10E-08	0.4373	0.0258	0.0113	0.0227
Yes	Yes	rs6815814	4		A	C	0.2525	0.052	0.0072	6.13E-13	0.2543	0.055	0.0086	1.59E-10	0.2539	0.0371	0.0132	0.004896
Yes	Yes	rs6828523	4	ADAM29	C	A	0.1175	-0.1019	0.0098	1.79E-25	0.1174	-0.1318	0.0118	5.45E-29	0.1175	-0.0017	0.0175	0.9247
Yes		rs77528541	4		G	T	0.1361	-0.0583	0.0096	1.41E-09	0.1358	-0.0545	0.0115	2.04E-06	0.1358	-0.0776	0.0178	1.31E-05
Yes	Yes	rs9790517	4	TET2	C	T	0.2264	0.0483	0.0073	5.03E-11	0.2271	0.0559	0.0087	1.59E-10	0.2277	0.0125	0.0134	0.3527
Yes	Yes	rs10472076	5		T	C	0.3766	0.0364	0.0064	9.64E-09	0.3767	0.034	0.0076	6.96E-06	0.3766	0.043	0.0115	0.000197
Yes	Yes	rs10474352	5		C	T	0.1594	-0.0586	0.0089	4.52E-11	0.1596	-0.0707	0.0107	3.39E-11	0.1597	-0.0162	0.016	0.3126
Yes	Yes	rs10941679	5		A	G	0.2522	0.1278	0.0071	5.61E-73	0.2511	0.1563	0.0084	8.27E-78	0.2521	0.0336	0.013	0.009429
Yes		rs116095464	5	AHRR	T	C	0.0544	0.0804	0.0136	3.80E-09	0.0543	0.0772	0.0161	1.68E-06	0.0553	0.0844	0.0243	0.000518
Yes		rs13162653	5	LOC401176	G	T	0.4555	-0.0321	0.0064	5.42E-07	0.4548	-0.0287	0.0076	0.000166	0.4562	-0.0323	0.0116	0.005479
Yes	Yes	rs1432679	5	EBF1	C	T	0.5663	-0.0717	0.0062	6.63E-31	0.5669	-0.0695	0.0074	5.77E-21	0.5656	-0.073	0.0113	1.06E-10
Yes	Yes	rs2012709	5	SUB1	C	T	0.4742	0.0358	0.0063	1.15E-08	0.475	0.039	0.0075	1.85E-07	0.4763	-0.0001	0.0114	0.9913
Yes		rs4562056	5		G	T	0.3323	0.0416	0.0067	4.72E-10	0.3314	0.0451	0.0079	1.43E-08	0.3322	0.023	0.0122	0.05887
Yes		rs62355902	5		A	T	0.1589	0.1734	0.0083	6.78E-98	0.1587	0.2032	0.0098	2.88E-96	0.1587	0.0722	0.0152	2.14E-06
Yes		rs6596100	5	HSPA4	C	T	0.2427	-0.0439	0.0076	7.74E-09	0.2434	-0.0592	0.0091	7.68E-11	0.2421	-0.0214	0.0138	0.1197
Yes		rs6882649	5	NREP	G	T	0.6651	0.0388	0.0066	3.67E-09	0.6645	0.0351	0.0078	7.77E-06	0.6643	0.0319	0.012	0.007875
Yes		rs7707921	5	ATG10	T	A	0.7541	0.0513	0.0073	1.69E-12	0.7549	0.0595	0.0087	8.09E-12	0.7542	0.032	0.0132	0.01528
Yes	Yes	rs11242675	6		C	T	0.6263	0.0249	0.0064	0.000101	0.6268	0.02	0.0076	0.00876	0.6281	0.0345	0.0117	0.003252

Yes		rs12207986	6	BCKDHB	G	A	0.5362	0.0375	0.0062	1.45E-09	0.536	0.0297	0.0074	6.13E-05	0.5356	0.0388	0.0113	0.000608
Yes	Yes	rs17529111	6		T	C	0.2212	0.045	0.0074	1.29E-09	0.2222	0.0328	0.0089	0.000249	0.2216	0.0646	0.0134	1.56E-06
Yes		rs2223621	6	CDKAL1	T	C	0.6162	-0.0408	0.0065	3.04E-10	0.6162	-0.0425	0.0077	3.54E-08	0.6152	-0.0148	0.0118	0.2103
Yes	Yes	rs2747652	6	ESR1	T	C	0.5263	0.0663	0.0062	1.31E-26	0.5264	0.053	0.0074	8.29E-13	0.526	0.0991	0.0113	1.93E-18
Yes	Yes	rs6569648	6	L3MBTL 3	C	T	0.7643	0.0512	0.0073	2.98E-12	0.7646	0.0468	0.0088	8.68E-08	0.7651	0.0741	0.0135	4.26E-08
Yes	Yes	rs9348512	6	MIR5689 HG	C	A	0.3341	0.0017	0.0066	0.7967	0.3343	0.0017	0.0078	0.8275	0.3352	0.0055	0.0119	0.6469
Yes	Yes	rs9485372	6	TAB2	G	A	0.1854	-0.0371	0.008	3.49E-06	0.1856	-0.0497	0.0096	2.03E-07	0.186	-0.0192	0.0146	0.187
Yes	Yes	rs17156577	7	CREB5	T	C	0.1121	0.0578	0.0098	4.25E-09	0.1121	0.0529	0.0117	6.11E-06	0.1125	0.0643	0.0179	0.000333
Yes	Yes	rs4593472	7	LINC-PINT	C	T	0.3529	-0.0438	0.0065	1.75E-11	0.3529	-0.0455	0.0077	4.15E-09	0.3514	-0.0311	0.0119	0.009171
Yes	Yes	rs6964587	7	AKAP9	G	T	0.3898	0.0409	0.0063	8.95E-11	0.3901	0.0452	0.0075	1.78E-09	0.3905	0.0231	0.0115	0.04452
Yes	Yes	rs720475	7	ARHGEF 5	G	A	0.2527	-0.0488	0.0072	1.20E-11	0.2525	-0.0508	0.0086	3.76E-09	0.2529	-0.0003	0.0132	0.9834
Yes	Yes	rs7971	7	DNAH11/ CDCA7L	A	G	0.3531	-0.0365	0.0065	1.93E-08	0.3525	-0.0353	0.0077	5.04E-06	0.3518	-0.0314	0.0119	0.008268
Yes	Yes	rs11780156	8		C	T	0.1678	0.0606	0.0082	1.09E-13	0.1674	0.0621	0.0097	1.72E-10	0.1693	0.0491	0.0147	0.000847
Yes		rs13267382	8	LINC005 36	A	G	0.6493	-0.0437	0.0065	1.60E-11	0.6478	-0.0427	0.0077	2.93E-08	0.649	-0.0369	0.0118	0.001786
Yes	Yes	rs13281615	8	CASC21 / CASC8	A	G	0.4049	0.1001	0.0063	1.89E-57	0.4042	0.1079	0.0075	2.34E-47	0.4062	0.0507	0.0114	8.82E-06
Yes	Yes	rs13365225	8		A	G	0.1752	-0.0767	0.0082	1.39E-20	0.1754	-0.0712	0.0098	4.57E-13	0.1771	-0.0963	0.015	1.44E-10
Yes	Yes	rs514192	8	LOC107 986961	A	T	0.6752	-0.0383	0.0066	5.61E-09	0.675	-0.0448	0.0078	9.86E-09	0.6755	-0.016	0.012	0.1818
Yes	Yes	rs6472903	8		G	T	0.8261	0.0778	0.0083	4.43E-21	0.8265	0.0784	0.0099	2.01E-15	0.8264	0.0439	0.0151	0.003656
Yes	Yes	rs9693444	8		A	C	0.6757	-0.0626	0.0066	1.60E-21	0.6755	-0.0683	0.0078	2.61E-18	0.6752	-0.0408	0.012	0.000638
Yes	Yes	rs1011970	9	CDKN2B -AS1	G	T	0.1653	0.066	0.0082	1.03E-15	0.1655	0.0576	0.0098	3.97E-09	0.166	0.0748	0.0148	4.67E-07
Yes	Yes	rs10759243	9		C	A	0.2889	0.0595	0.0068	2.23E-18	0.2902	0.0732	0.0081	1.10E-19	0.2883	0.0278	0.0125	0.02572
Yes	Yes	rs10760444	9	LMX1B	G	A	0.5667	-0.0358	0.0062	9.06E-09	0.5669	-0.0269	0.0074	0.000282	0.5667	-0.0493	0.0114	1.43E-05
Yes	Yes	rs10816625	9	LOC105 376214	A	G	0.0623	0.1094	0.0126	5.04E-18	0.062	0.1235	0.015	1.51E-16	0.0627	0.0689	0.0234	0.00319
Yes		rs10995201	10	ZNF365	A	G	0.1591	-0.1317	0.0087	1.56E-51	0.1591	-0.1379	0.0105	1.12E-39	0.1582	-0.0963	0.0159	1.57E-09
Yes	Yes	rs11199914	10		C	T	0.3209	-0.0456	0.0066	6.45E-12	0.3212	-0.0582	0.0079	2.44E-13	0.3212	-0.0045	0.012	0.707

Yes	Yes	rs2380205	10		C	T	0.4414	-0.0234	0.0062	0.000172	0.4413	-0.0261	0.0074	0.000443	0.4411	-0.0137	0.0113	0.2244
Yes	Yes	rs2981578	10	FGFR2	C	T	0.5323	-0.2084	0.0062	1.31E-245	0.5323	-0.2503	0.0074	6.37E-249	0.5307	-0.0383	0.0113	0.000705
Yes	Yes	rs704010	10	ZMIZ1	T	C	0.6203	-0.0787	0.0063	1.73E-35	0.6209	-0.0824	0.0075	1.02E-27	0.6207	-0.0504	0.0115	1.27E-05
Yes	Yes	rs7072776	10	LOC107 984214 /MLLT10	A	G	0.7124	-0.0618	0.0068	1.75E-19	0.7122	-0.0754	0.0082	2.78E-20	0.7119	0.0193	0.0127	0.1279
Yes	Yes	rs11820646	11		T	C	0.5959	0.0482	0.0063	2.11E-14	0.5961	0.0442	0.0075	3.97E-09	0.5971	0.0596	0.0115	2.39E-07
Yes	Yes	rs3903072	11		G	T	0.4727	-0.0434	0.0062	2.25E-12	0.4732	-0.0483	0.0074	5.94E-11	0.473	-0.0253	0.0112	0.02474
Yes	Yes	rs6597981	11		A	G	0.5178	0.0439	0.0062	1.35E-12	0.5179	0.0381	0.0074	2.49E-07	0.5182	0.0499	0.0113	9.86E-06
Yes	Yes	rs12422552	12		G	C	0.2589	0.0552	0.007	3.62E-15	0.2583	0.0483	0.0084	7.34E-09	0.2578	0.0555	0.0128	1.34E-05
Yes	Yes	rs1292011	12	LOC105 370003	A	G	0.4173	-0.0822	0.0063	4.39E-39	0.4166	-0.0994	0.0075	5.61E-40	0.4173	-0.0209	0.0114	0.06778
Yes	Yes	rs17356907	12		A	G	0.2985	-0.0898	0.0068	1.02E-39	0.2987	-0.0899	0.0081	1.69E-28	0.2979	-0.0694	0.0124	2.29E-08
	Yes	rs202049448	12		T	C	0.3376	-0.0387	0.007	2.69E-08	0.3389	-0.0353	0.0083	2.02E-05	0.3369	-0.0494	0.0128	0.00011
Yes	Yes	rs206966	12		C	T	0.1566	0.0487	0.0089	3.79E-08	0.1567	0.0506	0.0105	1.35E-06	0.1559	0.0487	0.0161	0.002475
Yes	Yes	rs7297051	12		C	T	0.2402	-0.1204	0.0074	2.95E-60	0.2407	-0.1102	0.0088	3.25E-36	0.2398	-0.14	0.0136	8.49E-25
Yes	Yes	rs11571833	13	BRCA2	A	T	0.0081	0.2727	0.0346	3.10E-15	0.0081	0.239	0.0397	1.69E-09	0.0081	0.4346	0.0588	1.49E-13
Yes	Yes	rs6562760	13		A	G	0.766	0.0443	0.0073	1.49E-09	0.7663	0.0412	0.0087	2.44E-06	0.7656	0.0826	0.0135	8.69E-10
Yes	Yes	rs11627032	14	RIN3	T	C	0.2572	-0.0481	0.0073	4.11E-11	0.2569	-0.0438	0.0088	6.72E-07	0.2566	-0.0626	0.0133	2.42E-06
Yes	Yes	rs2236007	14	LOC105 370455 /PAX9	G	A	0.2113	-0.0719	0.0076	4.17E-21	0.2114	-0.0801	0.0091	1.93E-18	0.2112	-0.0368	0.0139	0.008037
Yes	Yes	rs2588809	14	RAD51B	T	C	0.8378	-0.0628	0.0084	6.34E-14	0.8376	-0.0742	0.01	9.00E-14	0.8371	0.0047	0.0155	0.7605
Yes	Yes	rs941764	14	CCDC88 C	A	G	0.3429	0.0463	0.0065	8.21E-13	0.343	0.0477	0.0077	6.37E-10	0.3434	0.0186	0.0118	0.1162
Yes	Yes	rs2290203	15	PRC1- AS1/ PRC1	G	A	0.204	-0.0474	0.0077	8.07E-10	0.2045	-0.0475	0.0092	2.35E-07	0.204	-0.0494	0.0141	0.000471
Yes	Yes	rs13329835	16	CDYL2	A	G	0.2243	0.0786	0.0073	8.75E-27	0.225	0.0823	0.0087	2.88E-21	0.2252	0.0426	0.0133	0.001351
Yes	Yes	rs17817449	16	FTO	T	G	0.4054	-0.0599	0.0063	2.52E-21	0.4062	-0.0564	0.0075	5.59E-14	0.4052	-0.0736	0.0115	1.80E-10
Yes		rs2432539	16	AMFR	A	G	0.5996	-0.0349	0.0064	4.02E-08	0.5993	-0.0364	0.0076	1.47E-06	0.5986	-0.0221	0.0115	0.05487
Yes	Yes	rs4496150	16	LOC105 371393	C	A	0.2466	-0.0416	0.0072	8.09E-09	0.2473	-0.042	0.0086	1.01E-06	0.2471	-0.0413	0.0131	0.001679
Yes	Yes	rs4784227	16	CASC16	C	T	0.2387	0.2153	0.0071	6.78E-201	0.2385	0.2296	0.0084	3.93E-164	0.2403	0.1368	0.0128	1.72E-26
Yes		rs2532263	17	KANSL1	G	A	0.182	-0.0605	0.0084	6.86E-13	0.1817	-0.0584	0.01	6.16E-09	0.1826	-0.0483	0.0154	0.001693

Yes		rs2787486	17	STXBP4	A	C	0.3002	-0.0758	0.0068	5.56E-29	0.2996	-0.0898	0.0081	2.09E-28	0.2997	-0.0379	0.0124	0.002164
Yes	Yes	rs745570	17		A	G	0.5044	-0.0389	0.0062	3.87E-10	0.5035	-0.0349	0.0074	2.44E-06	0.5046	-0.0501	0.0114	1.04E-05
Yes		rs117618124	18	GAREM1	T	C	0.0453	-0.1074	0.0156	5.46E-12	0.0452	-0.0907	0.0186	1.07E-06	0.0452	-0.1342	0.0294	5.04E-06
Yes	Yes	rs527616	18		C	G	0.6211	0.0499	0.0064	6.70E-15	0.6202	0.0515	0.0076	1.37E-11	0.6219	0.0178	0.0117	0.1268
Yes	Yes	rs6507583	18	SETBP1	A	G	0.0692	-0.087	0.0124	2.23E-12	0.0688	-0.1025	0.0149	6.14E-12	0.0691	-0.034	0.0225	0.1311
Yes	Yes	rs3760982	19	KCNN4	A	G	0.539	-0.051	0.0062	1.42E-16	0.5406	-0.0521	0.0074	1.53E-12	0.539	-0.0612	0.0112	5.34E-08
Yes		rs67397200	19		C	G	0.2971	0.0381	0.0067	1.61E-08	0.297	-0.0061	0.0081	0.454	0.2974	0.1543	0.0121	2.67E-37
Yes		rs78269692	19	NFIX	T	C	0.0488	0.0922	0.0154	1.92E-09	0.0481	0.0986	0.0183	7.12E-08	0.0493	0.0753	0.0275	0.00615
Yes	Yes	rs16991615	20	MCM8	G	A	0.0626	0.0758	0.0126	1.92E-09	0.0623	0.0723	0.0151	1.57E-06	0.0623	0.098	0.0228	1.72E-05
Yes	Yes	rs2284378	20	RALY	T	C	0.6822	-0.0142	0.0066	0.03201	0.6834	-0.0005	0.0079	0.9487	0.683	-0.0289	0.0121	0.01722
Yes		rs6122906	20		A	G	0.1816	0.0507	0.008	2.45E-10	0.1813	0.0473	0.0095	6.83E-07	0.1815	0.0435	0.0146	0.002897
Yes	Yes	rs2823093	21		G	A	0.2682	-0.0653	0.007	1.52E-20	0.2684	-0.0833	0.0084	4.15E-23	0.2676	-0.0069	0.0127	0.5862
Yes	Yes	rs132390	22	EMID1/ LOC105 372985	C	T	0.9633	-0.0945	0.0165	1.15E-08	0.9631	-0.0824	0.0195	2.35E-05	0.9635	-0.0507	0.031	0.1015
Yes		rs28512361	22	LOC107 985535	G	A	0.1127	0.0611	0.0109	2.25E-08	0.1124	0.062	0.0129	1.66E-06	0.1132	0.0853	0.0197	1.54E-05
Yes	Yes	rs738321	22	PLA2G6	C	G	0.3767	-0.0474	0.0064	1.04E-13	0.3763	-0.0632	0.0076	1.11E-16	0.3775	-0.0152	0.0116	0.1887

Supplementary Table 5. Genetic correlations between body mass index (BMI), breast size, overall breast cancer risk, ER-positive breast cancer risk and ER-negative breast cancer risk. Rg: Correlation coefficient.

	BMI			Breast size			BMI	Overall		ER-positive
	Overall	ER-positive	ER-negative	Overall	ER-positive	ER-negative	Breast size	ER-positive	ER-negative	ER-negative
Rg	-0.0518	-0.0332	-0.0412	0.0101	-0.008	0.0393	0.4983	0.9828	0.7512	0.6131
P-value	0.0849	0.2589	0.3268	0.8285	0.8642	0.5288	3.89E-43	0	3.09E-233	8.88E-88

Supplementary Figure 1. Odds ratios (ORs) and 95% confidence intervals (CI) for the association between breast size (exposure) and BMI (outcome) based on the different Mendelian randomization approaches used in this study. *Value based on causal effect estimate from MR-Egger regression; corresponding MR-Egger intercept value testing presence of directional (bias inducing) pleiotropy not shown.

Outcome	Method	P-value	OR (95%CI)
BMI	*MR Egger	0.195	1.17 (1.00 - 1.38)
	Inverse variance weighted	0.927	1.00 (0.95 - 1.05)
	Weighted median	0.354	1.02 (0.97 - 1.08)
	Weighted mode	0.428	1.03 (0.97 - 1.09)

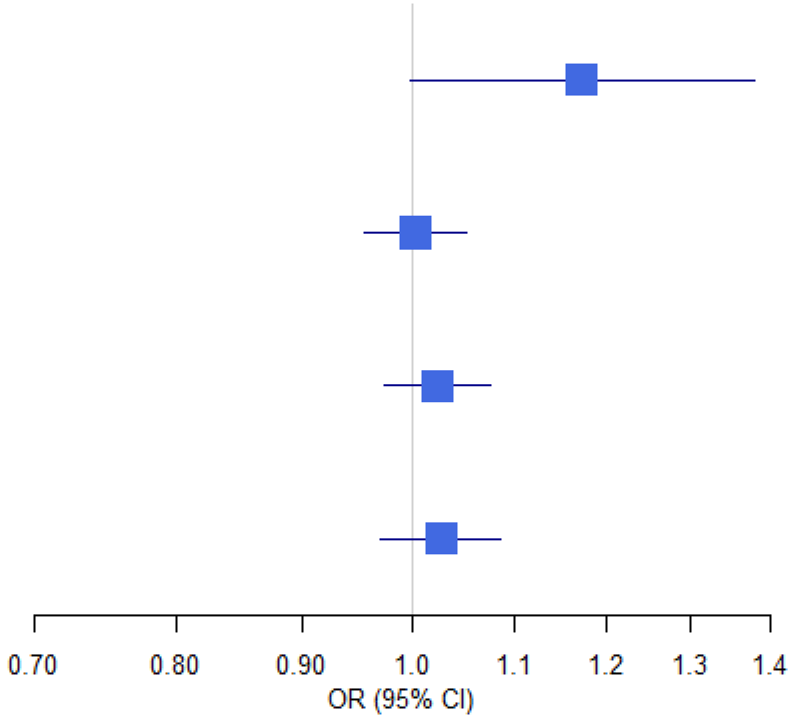

Supplementary Figure 2. **(A)** Scatter plot of SNP-BMI associations against SNP-breast size associations with estimates from different Mendelian randomization methods indicated by corresponding coloured lines. **(B)** Leave-one-out permutation analysis plot for breast size obtained by leaving out the SNP indicated and repeating the standard inverse-variance weighted method with the rest of the 3-SNP instrumental variable.

(A)

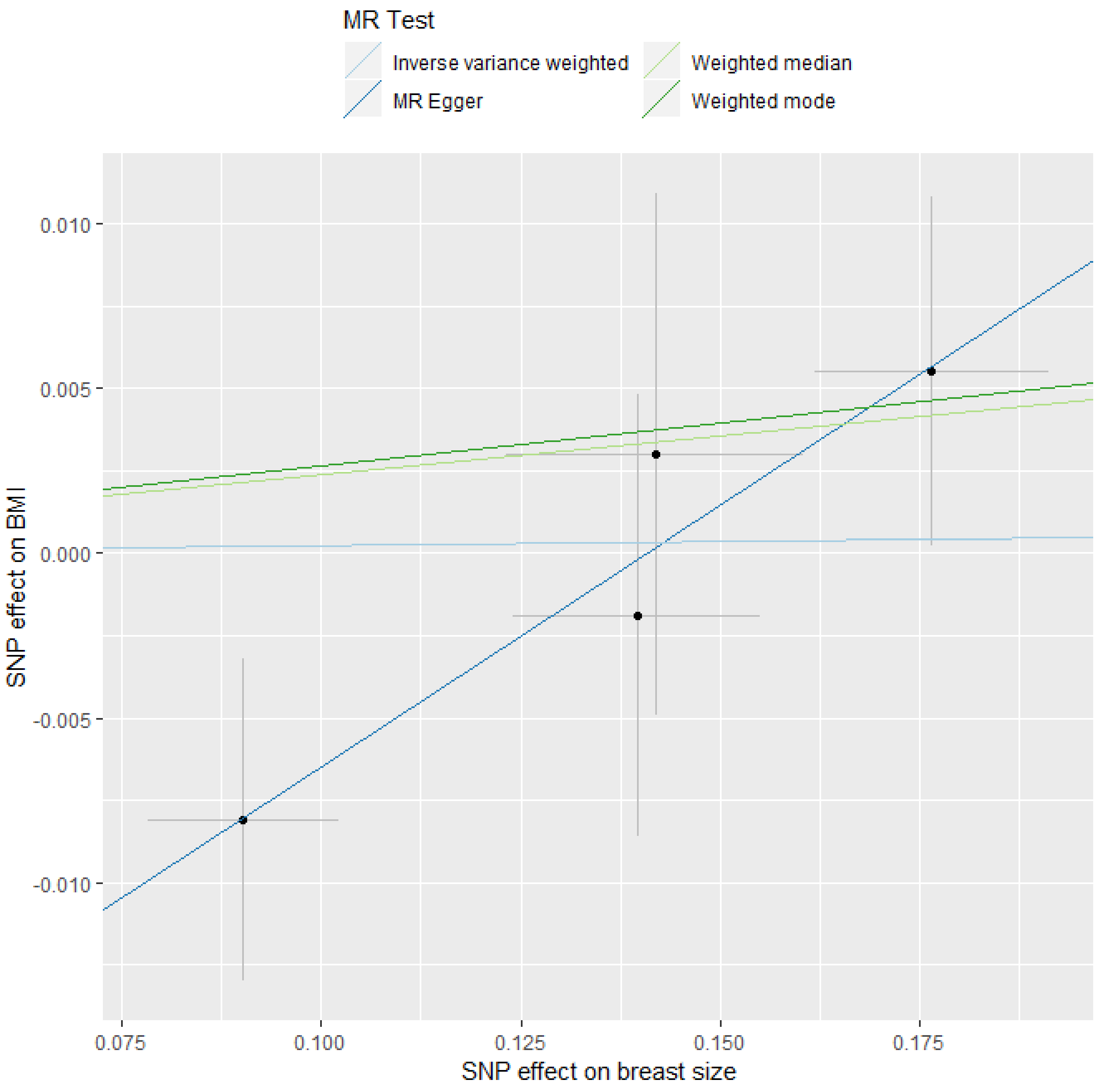

(B)

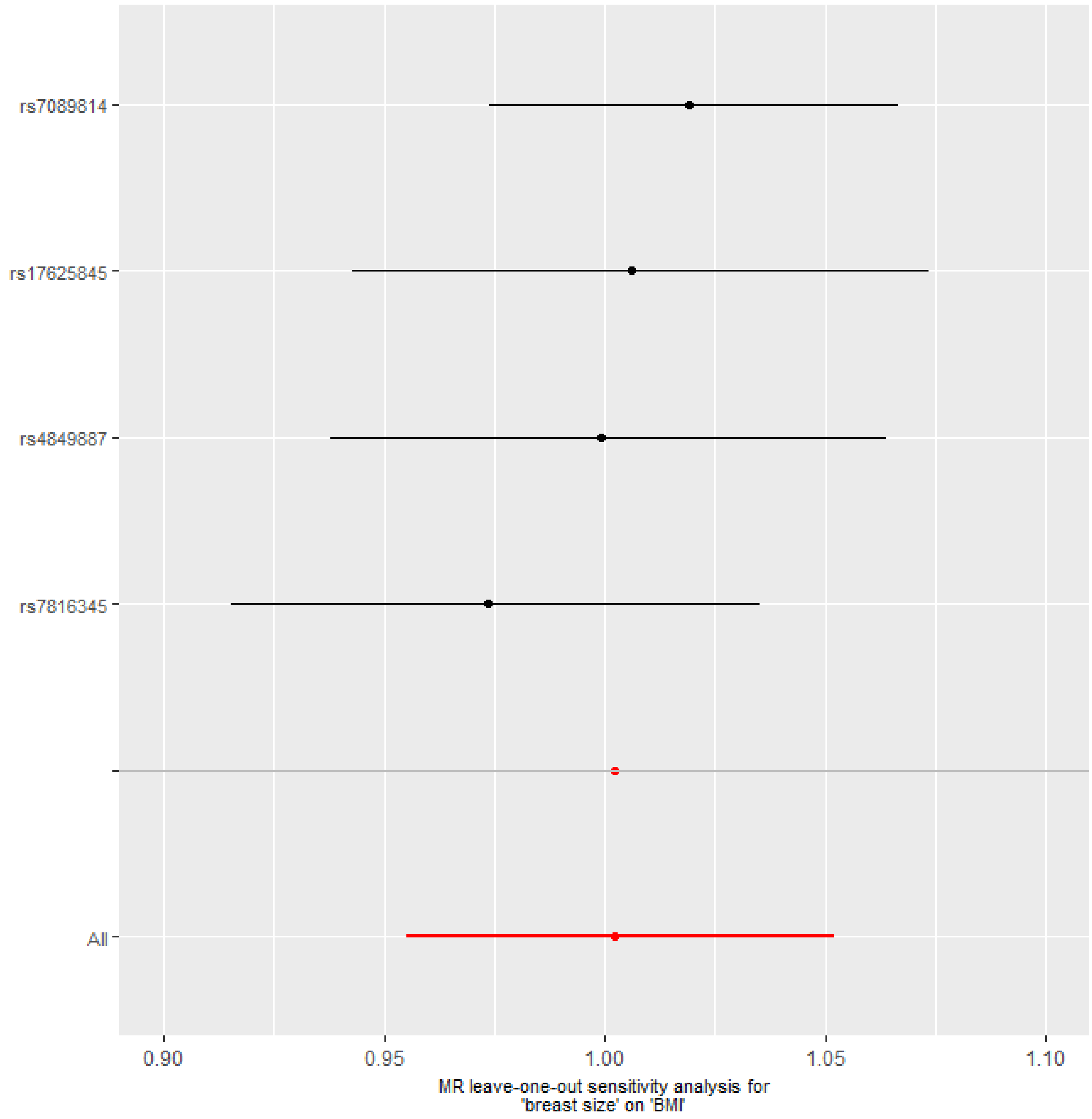

rs7089814
rs17625845
rs4849887
rs7816345
All
0.90
0.95
1.00
1.05
1.10
MR leave-one-out sensitivity analysis for
'breast size' on 'BMI'

Supplementary Figure 3. Odds ratios (ORs) and 95% confidence intervals (CI) for the association between overall breast cancer risk (exposure) and two outcomes (i.e. **(a)** breast size and **(b)** body mass index [BMI]) based on the different Mendelian randomization approaches used in this study. *Value based on causal effect estimate from MR-Egger regression; corresponding MR-Egger intercept value testing presence of directional (bias inducing) pleiotropy not shown.

Outcome	Method	P-value	OR (95%CI)
(a) Breast size	*MR Egger	0.763	0.97 (0.82 - 1.16)
	Inverse variance weighted	0.528	1.03 (0.94 - 1.12)
	Weighted median	0.752	0.99 (0.92 - 1.06)
	Weighted mode	0.675	1.03 (0.91 - 1.16)
(b) BMI	*MR Egger	0.880	1.01 (0.93 - 1.09)
	Inverse variance weighted	0.311	0.98 (0.94 - 1.02)
	Weighted median	0.823	1.00 (0.97 - 1.03)
	Weighted mode	0.900	1.00 (0.97 - 1.03)

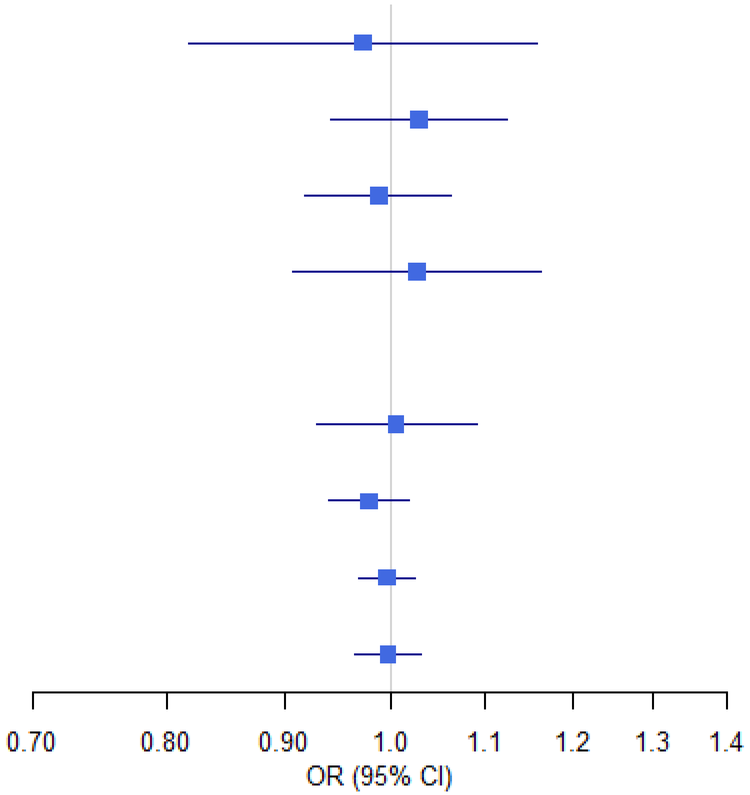

Supplementary Figure 4. Odds ratios (ORs) and 95% confidence intervals (CI) for the association between ER-positive breast cancer risk (exposure) and two outcomes (i.e. **(a)** breast size and **(b)** body mass index [BMI]) based on the different Mendelian randomization approaches used in this study. *Value based on causal effect estimate from MR-Egger regression; corresponding MR-Egger intercept value testing presence of directional (bias inducing) pleiotropy not shown.

Outcome	Method	P-value	OR (95%CI)
(a) Breast size	*MR Egger	0.752	0.98 (0.84 - 1.13)
	Inverse variance weighted	0.586	1.02 (0.94 - 1.11)
	Weighted median	0.663	0.99 (0.92 - 1.05)
	Weighted mode	0.931	0.99 (0.70 - 1.38)
(b) BMI	*MR Egger	0.887	1.01 (0.94 - 1.08)
	Inverse variance weighted	0.389	0.98 (0.95 - 1.02)
	Weighted median	0.831	1.00 (0.97 - 1.02)
	Weighted mode	0.911	0.99 (0.78 - 1.24)

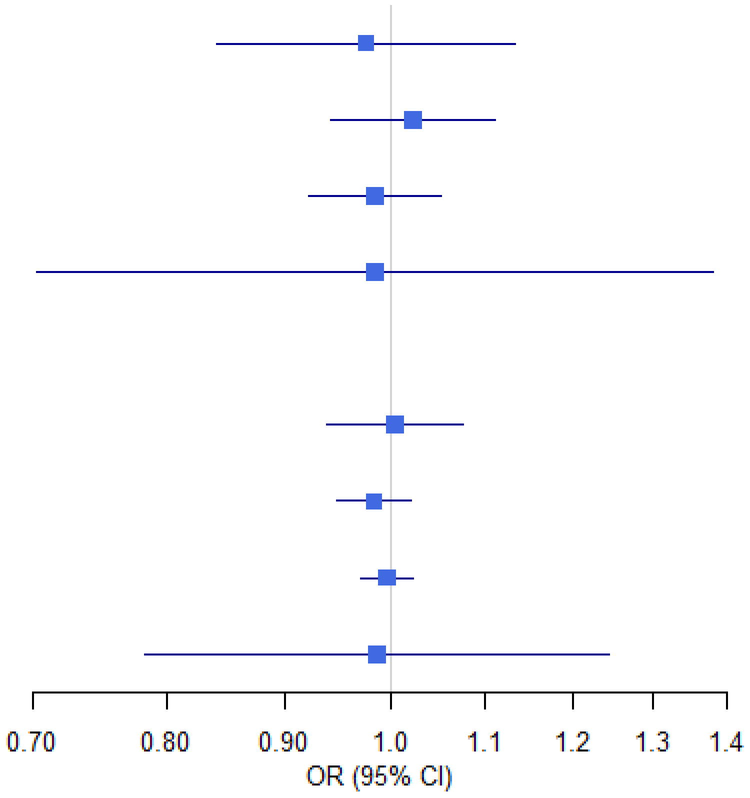

Supplementary Figure 5. Odds ratios (ORs) and 95% confidence intervals (CI) for the association between ER-negative breast cancer risk (exposure) and two outcomes (i.e. **(a)** breast size and **(b)** body mass index [BMI]) based on the different Mendelian randomization approaches used in this study. *Value based on causal effect estimate from MR-Egger regression; corresponding MR-Egger intercept value testing presence of directional (bias inducing) pleiotropy not shown.

Outcome	Method	P-value	OR (95%CI)
(a) Breast size	*MR Egger	0.724	1.03 (0.86 - 1.25)
	Inverse variance weighted	0.479	1.04 (0.93 - 1.16)
	Weighted median	0.486	1.04 (0.94 - 1.15)
	Weighted mode	0.988	1.14 (0.00 - Inf)
(b) BMI	*MR Egger	0.644	0.98 (0.90 - 1.07)
	Inverse variance weighted	0.073	0.95 (0.91 - 1.00)
	Weighted median	0.929	1.00 (0.96 - 1.03)
	Weighted mode	0.999	1.00 (0.16 - 6.35)

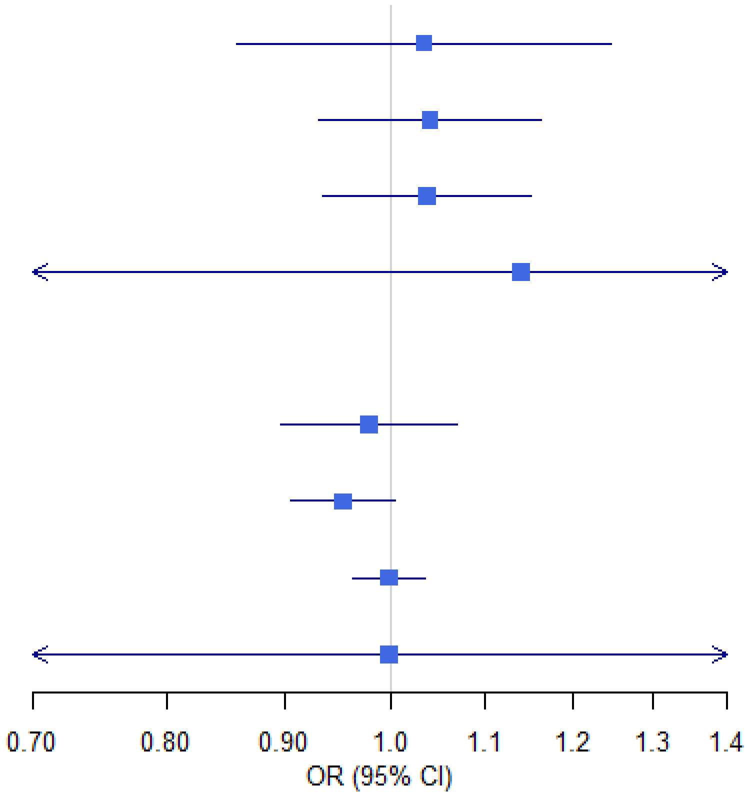

Supplementary Figure 6. Scatter plots of SNP-body mass index (BMI) associations against SNP-breast cancer risk associations for overall breast cancer **(A)**, estrogen receptor (ER)-positive breast cancer **(B)**, and ER-negative breast cancer **(C)** with estimates from different Mendelian randomization methods indicated by corresponding coloured lines). **(D-F)** Corresponding leave-one-out permutation analysis plots for BMI obtained by leaving out the SNP indicated and repeating the standard inverse-variance weighted method with the rest of the 80-SNP instrumental variables used.

(A)

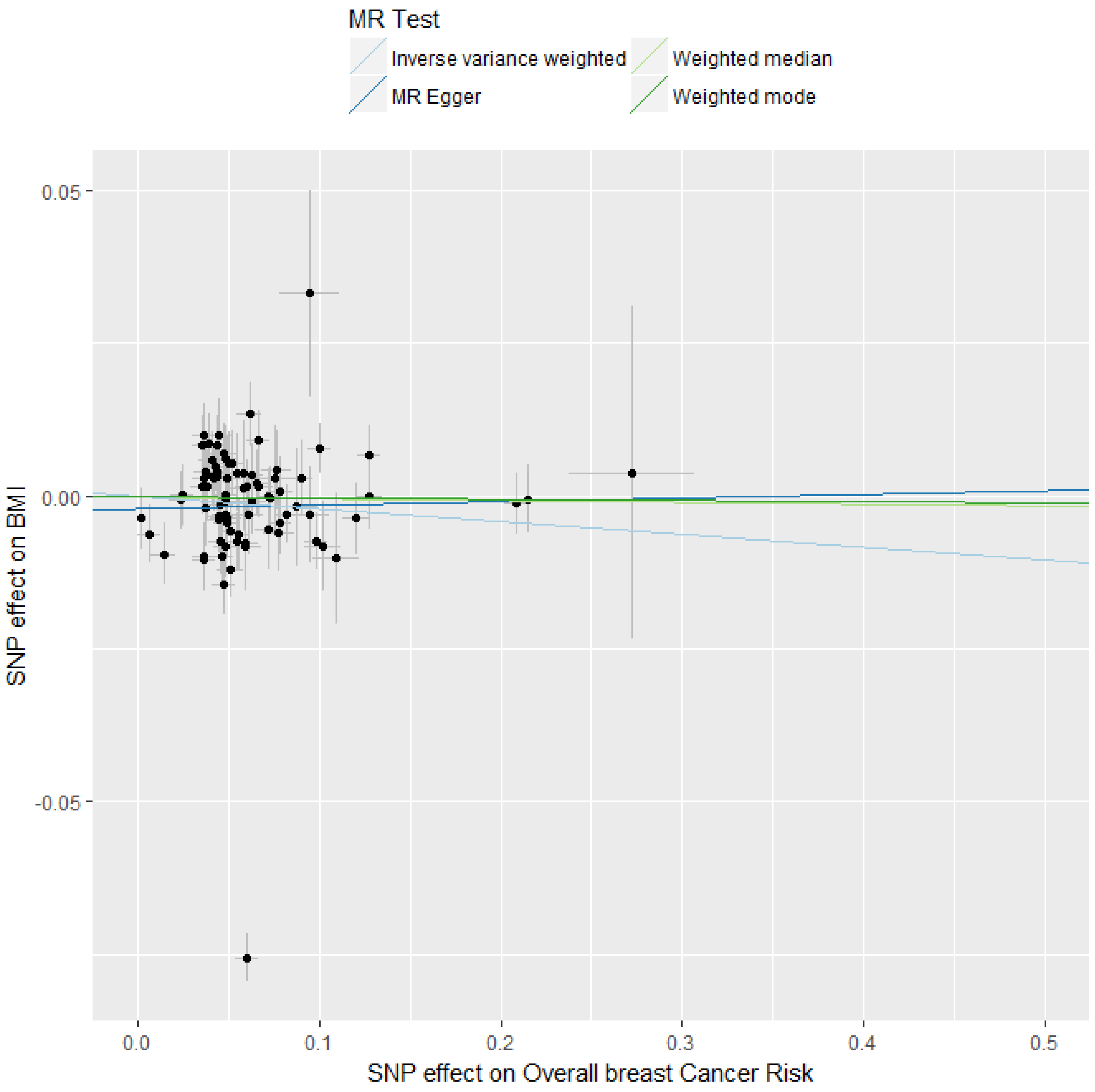

(B)

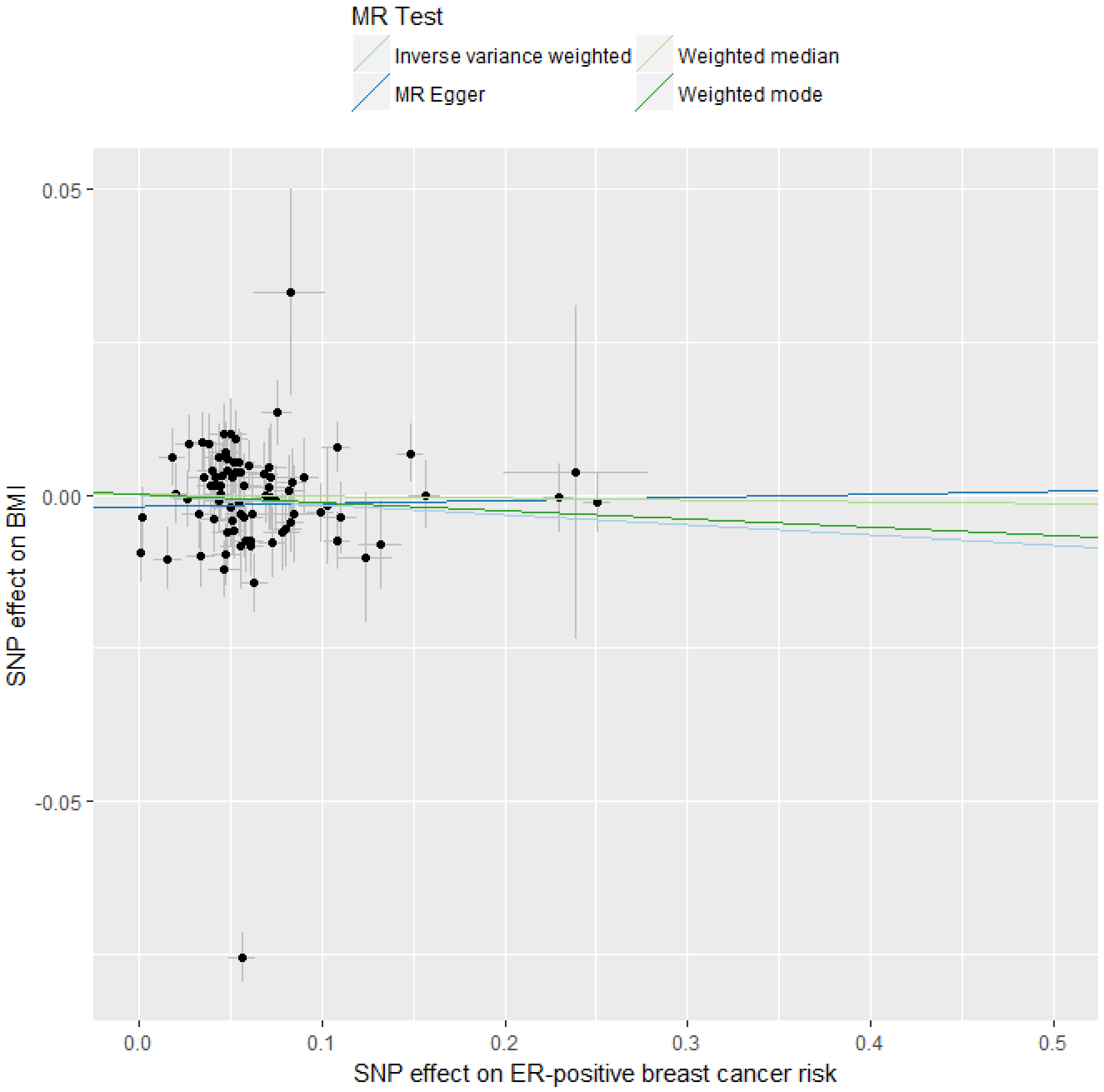
MR Test
Inverse variance weighted
Weighted median
MR Egger
Weighted mode
0.05
0.00
-0.05
0.0
0.1
0.2
0.3
0.4
0.5
SNP effect on BMI
SNP effect on ER-positive breast cancer risk

(C)

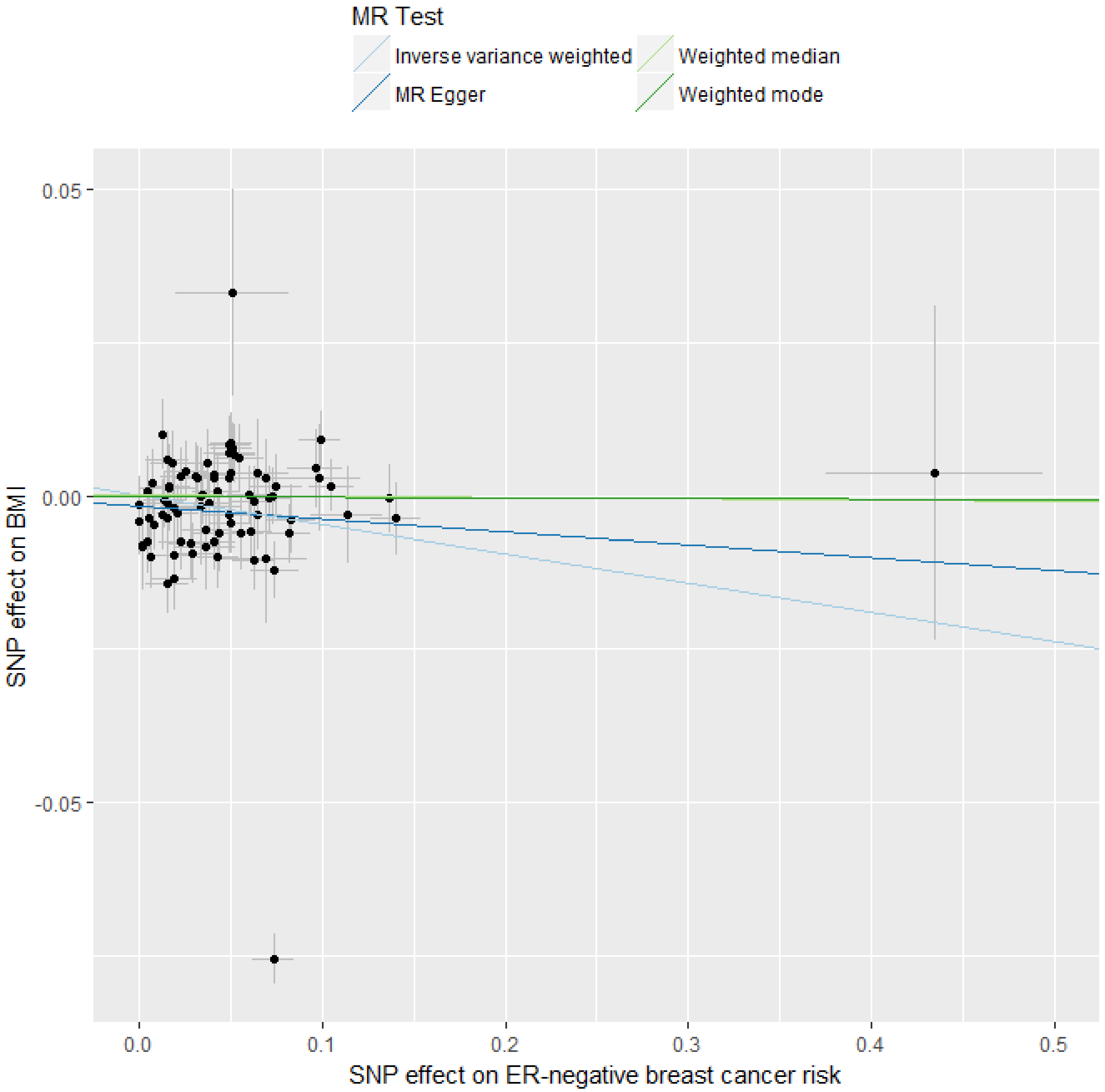
MR Test
Inverse variance weighted
Weighted median
MR Egger
Weighted mode
0.05
0.00
-0.05
SNP effect on BMI
0.0
0.1
0.2
0.3
0.4
0.5
SNP effect on ER-negative breast cancer risk

(D)

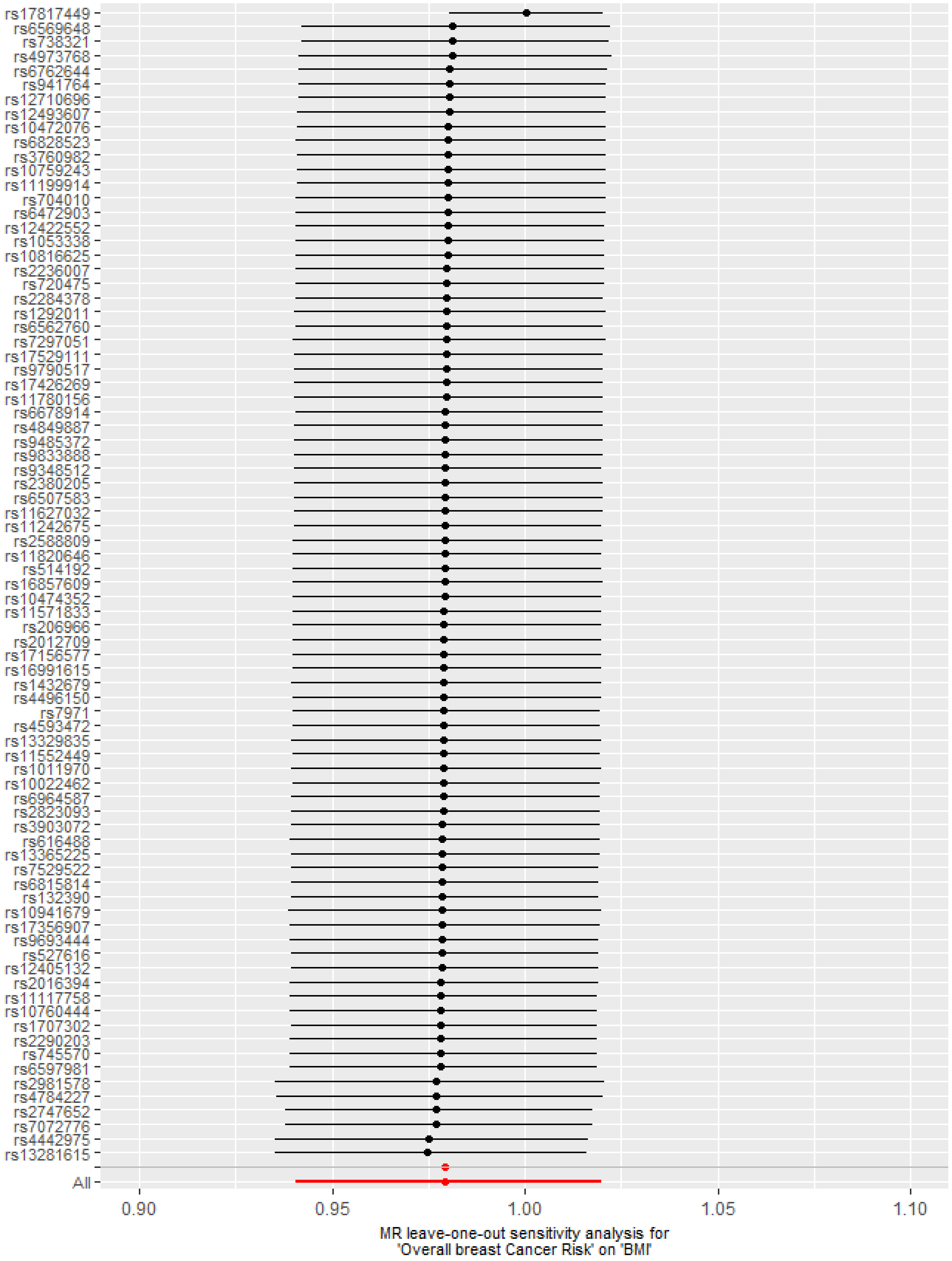
rs17817449
rs6569648
rs738321
rs4973768
rs6762644
rs941764
rs12710696
rs12493607
rs10472076
rs6828523
rs3760982
rs10759243
rs11199914
rs704010
rs6472903
rs12422552
rs1053338
rs10816625
rs2236007
rs720475
rs2284378
rs1292011
rs6562760
rs7297051
rs17529111
rs9790517
rs17426269
rs11780156
rs6678914
rs4849887
rs9485372
rs9833888
rs9348512
rs2380205
rs6507583
rs11627032
rs11242675
rs2588809
rs11820646
rs514192
rs16857609
rs10474352
rs11571833
rs206966
rs2012709
rs17156577
rs16991615
rs1432679
rs4496150
rs7971
rs4593472
rs13329835
rs11552449
rs1011970
rs10022462
rs6964587
rs2823093
rs3903072
rs616488
rs13365225
rs7529522
rs6815814
rs132390
rs10941679
rs17356907
rs9693444
rs527616
rs12405132
rs2016394
rs11117758
rs10760444
rs1707302
rs2290203
rs745570
rs6597981
rs2981578
rs4784227
rs2747652
rs7072776
rs4442975
rs13281615
All
0.90
0.95
1.00
1.05
1.10
MR leave-one-out sensitivity analysis for
'Overall breast Cancer Risk' on 'BMI'

(E)

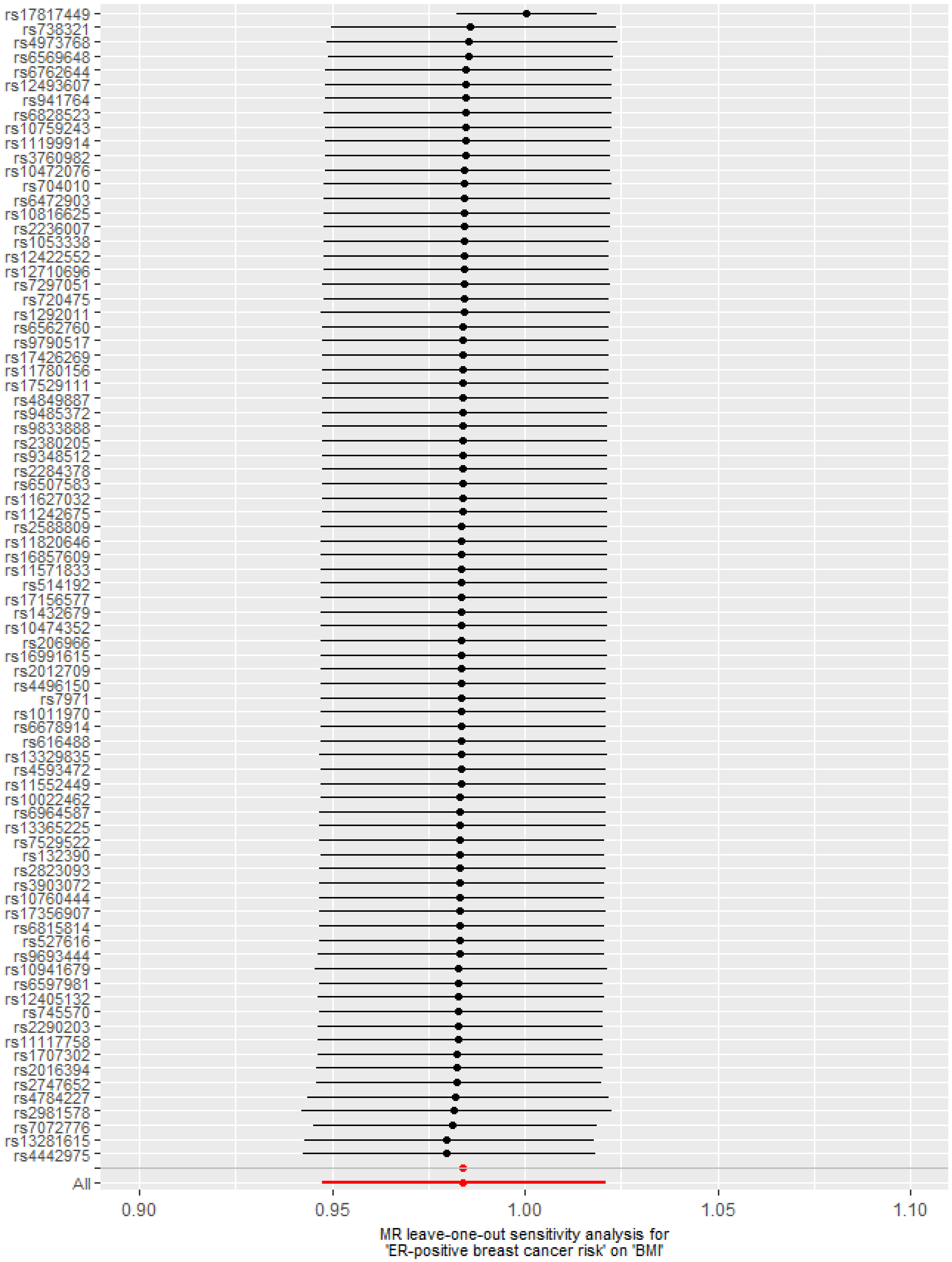
rs17817449
rs738321
rs4973768
rs6569648
rs6762644
rs12493607
rs941764
rs6828523
rs10759243
rs11199914
rs3760982
rs10472076
rs704010
rs6472903
rs10816625
rs2236007
rs1053338
rs12422552
rs12710696
rs7297051
rs720475
rs1292011
rs6562760
rs9790517
rs17426269
rs11780156
rs17529111
rs4849887
rs9485372
rs9833888
rs2380205
rs9348512
rs2284378
rs6507583
rs11627032
rs11242675
rs2588809
rs11820646
rs16857609
rs11571833
rs514192
rs17156577
rs1432679
rs10474352
rs206966
rs16991615
rs2012709
rs4496150
rs7971
rs1011970
rs6678914
rs616488
rs13329835
rs4593472
rs11552449
rs10022462
rs6964587
rs13365225
rs7529522
rs132390
rs2823093
rs3903072
rs10760444
rs17356907
rs6815814
rs527616
rs9693444
rs10941679
rs6597981
rs12405132
rs745570
rs2290203
rs11117758
rs1707302
rs2016394
rs2747652
rs4784227
rs2981578
rs7072776
rs13281615
rs4442975
All
0.90
0.95
1.00
1.05
1.10
MR leave-one-out sensitivity analysis for
'ER-positive breast cancer risk' on 'BMI'

(F)

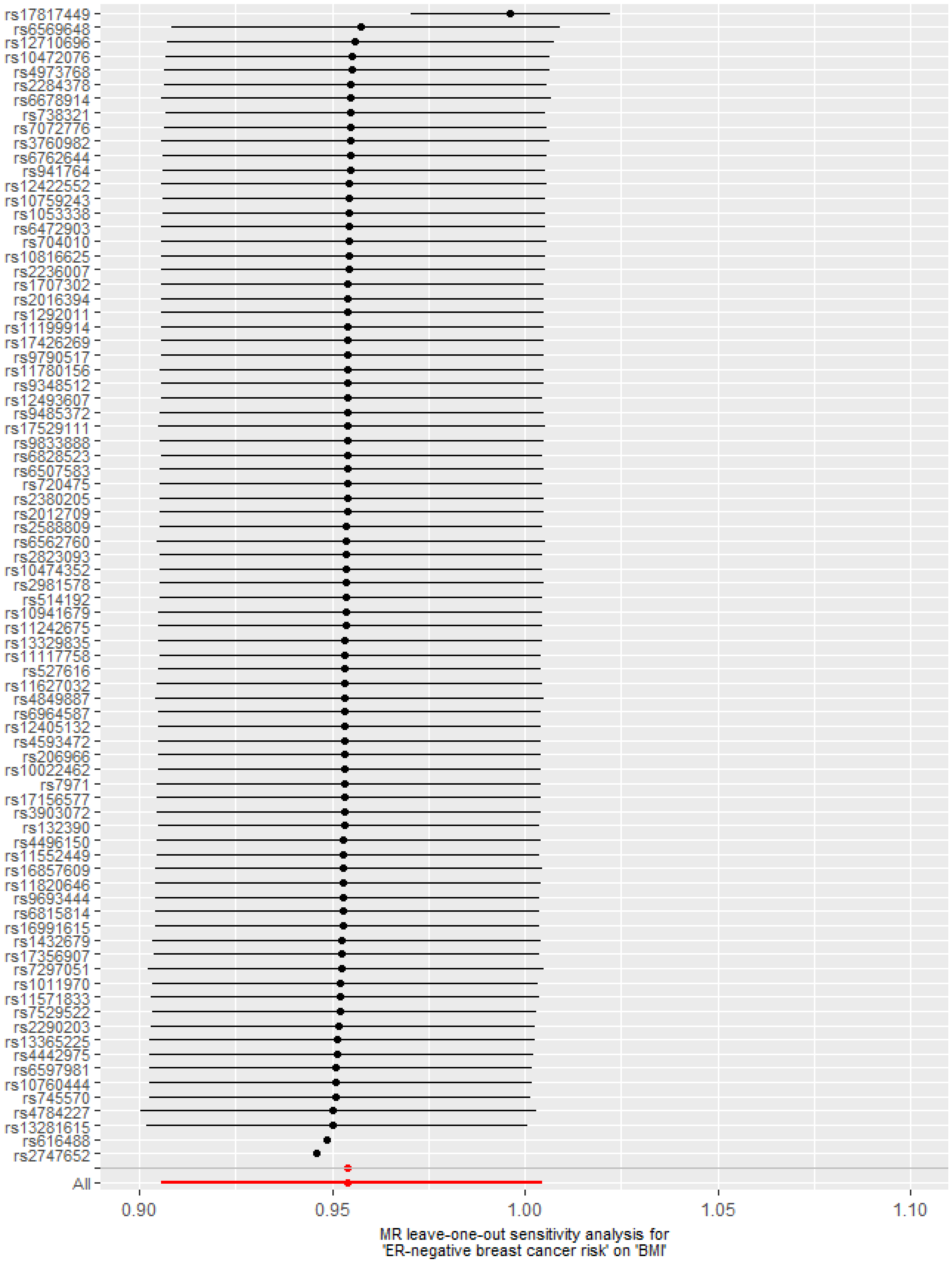
rs17817449
rs6569648
rs12710696
rs10472076
rs4973768
rs2284378
rs6678914
rs738321
rs7072776
rs3760982
rs6762644
rs941764
rs12422552
rs10759243
rs1053338
rs6472903
rs704010
rs10816625
rs2236007
rs1707302
rs2016394
rs1292011
rs11199914
rs17426269
rs9790517
rs11780156
rs9348512
rs12493607
rs9485372
rs17529111
rs9833888
rs6828523
rs6507583
rs720475
rs2380205
rs2012709
rs2588809
rs6562760
rs2823093
rs10474352
rs2981578
rs514192
rs10941679
rs11242675
rs13329835
rs11117758
rs527616
rs11627032
rs4849887
rs6964587
rs12405132
rs4593472
rs206966
rs10022462
rs7971
rs17156577
rs3903072
rs132390
rs4496150
rs11552449
rs16857609
rs11820646
rs9693444
rs6815814
rs16991615
rs1432679
rs17356907
rs7297051
rs1011970
rs11571833
rs7529522
rs2290203
rs13365225
rs4442975
rs6597981
rs10760444
rs745570
rs4784227
rs13281615
rs616488
rs2747652
All
0.90
0.95
1.00
1.05
1.10
MR leave-one-out sensitivity analysis for
'ER-negative breast cancer risk' on 'BMI'

Supplementary Figure 7. Scatter plots of SNP-breast size associations against SNP-breast cancer risk (**[A]**, overall, **[B]**, ER-positive and **[C]**, ER-negative) associations with estimates from different Mendelian randomization methods indicated by corresponding coloured lines. **(D-F)** Leave-one-out permutation analysis plots for breast size obtained by leaving out the SNP indicated and repeating the standard inverse-variance weighted method with the rest of the 112-SNP instrumental variable used.

(A)

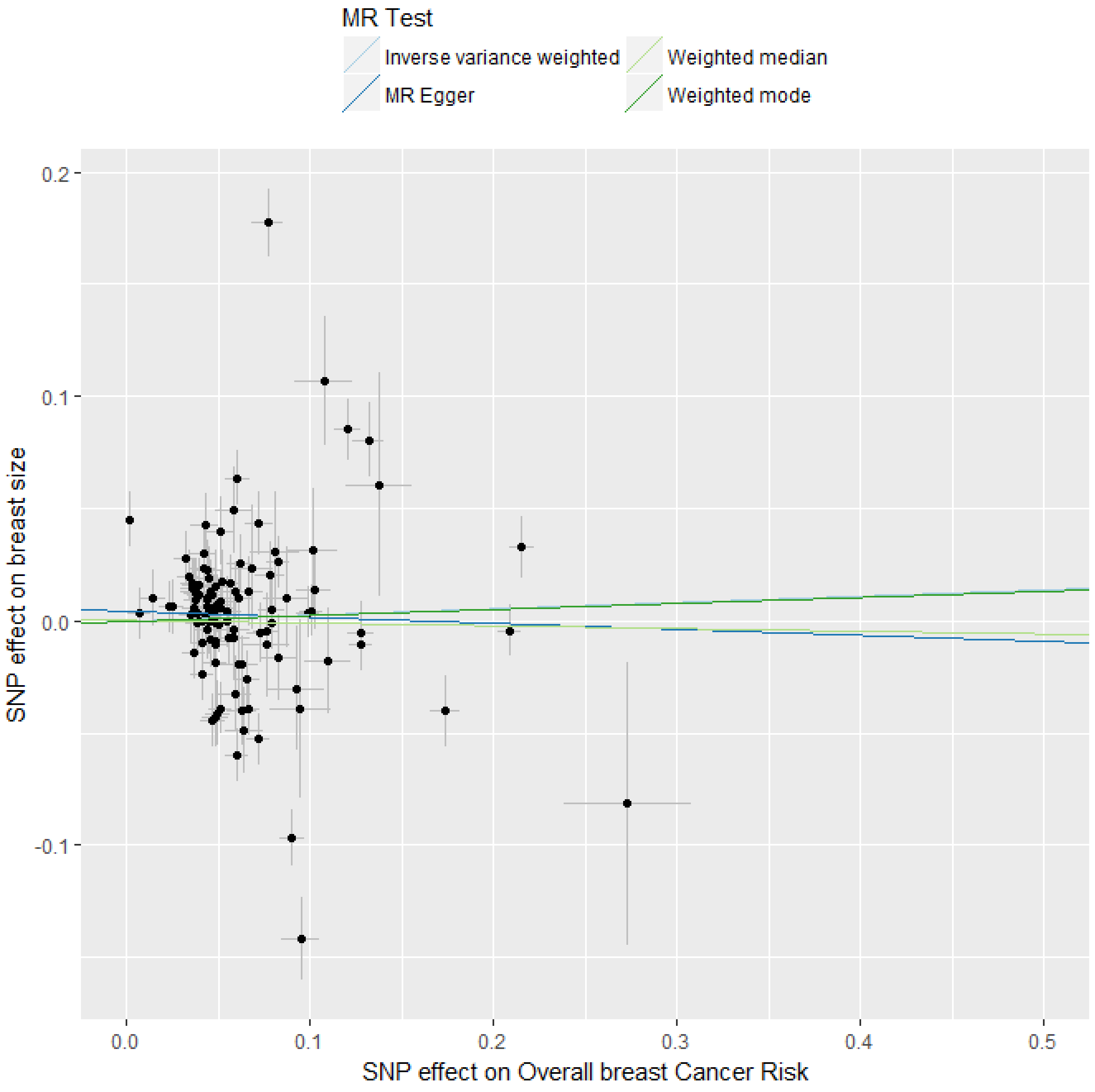

(B)

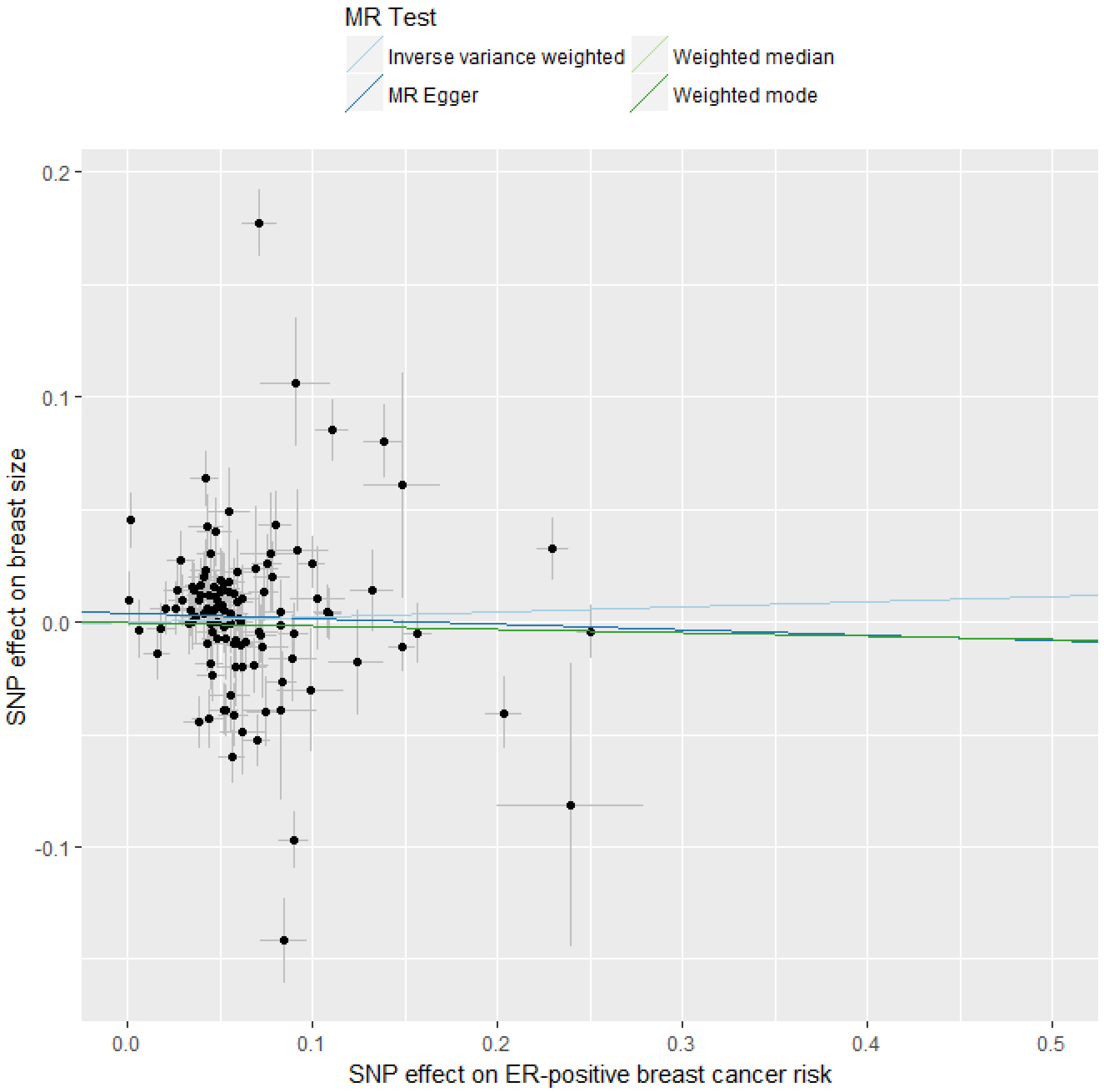
MR Test
Inverse variance weighted
Weighted median
MR Egger
Weighted mode
0.2
0.1
0.0
-0.1
SNP effect on breast size
0.0
0.1
0.2
0.3
0.4
0.5
SNP effect on ER-positive breast cancer risk

(C)

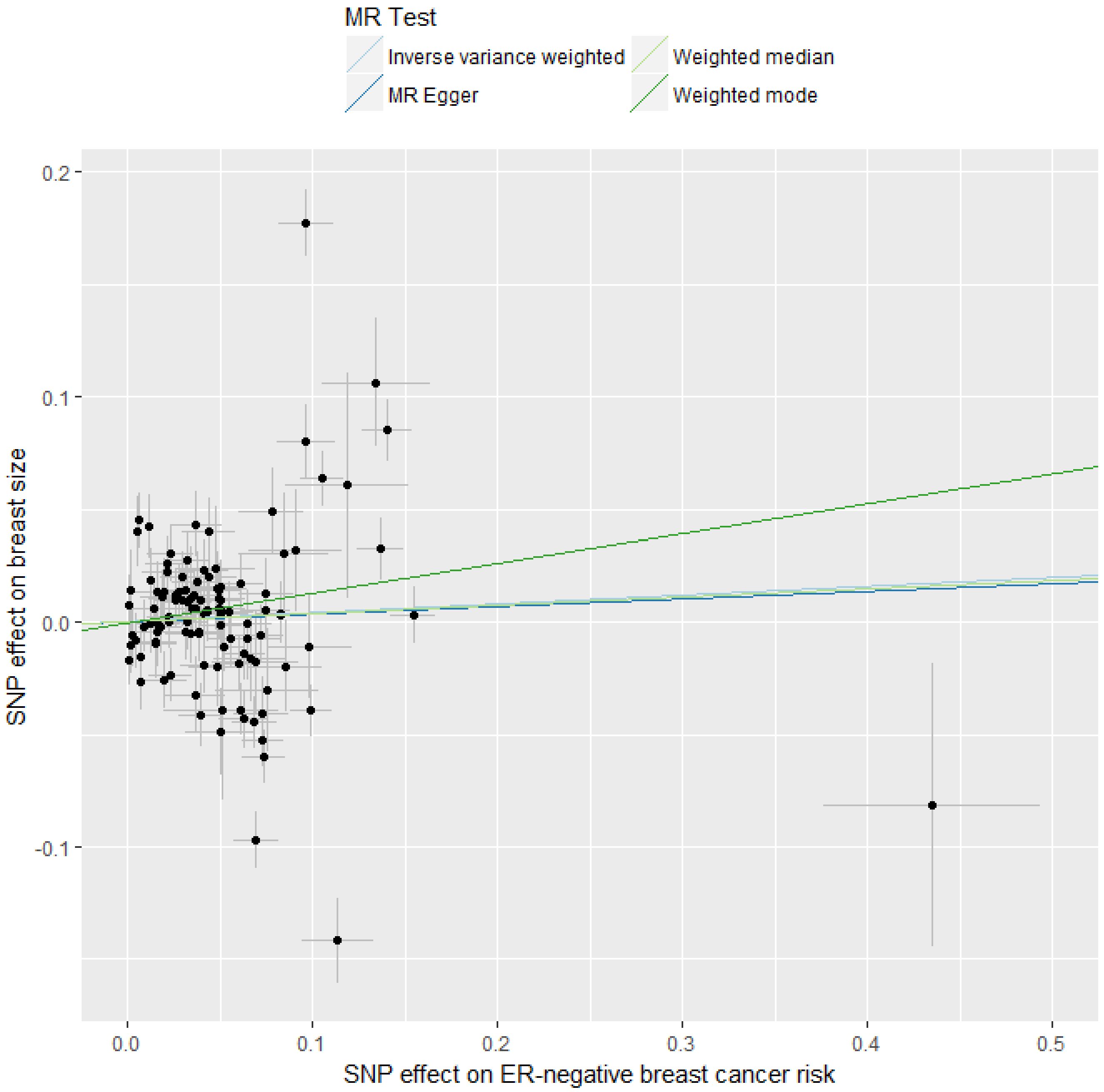
MR Test
Inverse variance weighted
Weighted median
MR Egger
Weighted mode
0.2
0.1
0.0
-0.1
SNP effect on breast size
0.0
0.1
0.2
0.3
0.4
0.5
SNP effect on ER-negative breast cancer risk

(D)

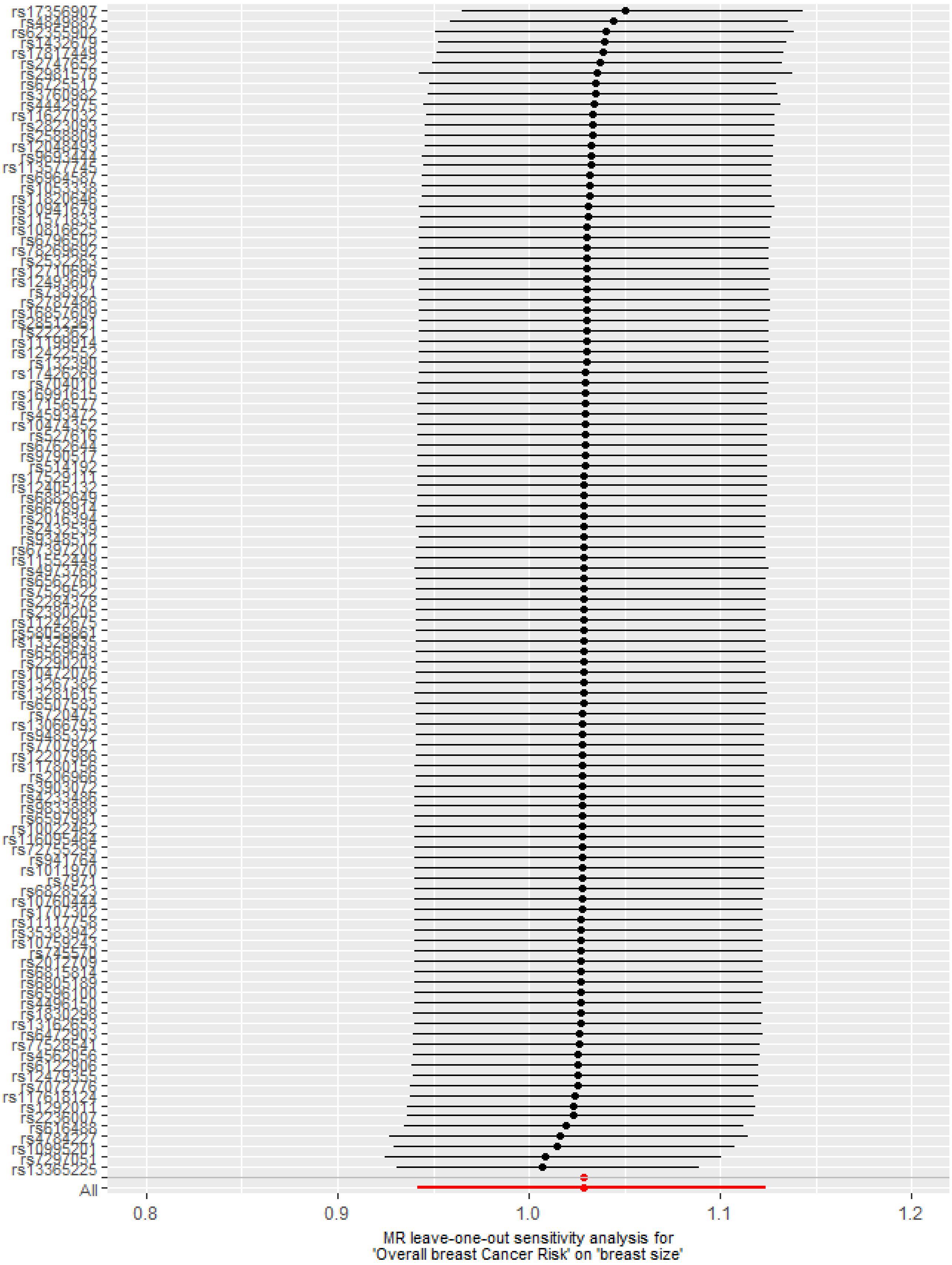

All
0.8
0.9
1.0
1.1
1.2
MR leave-one-out sensitivity analysis for
'Overall breast Cancer Risk' on 'breast size'

(E)

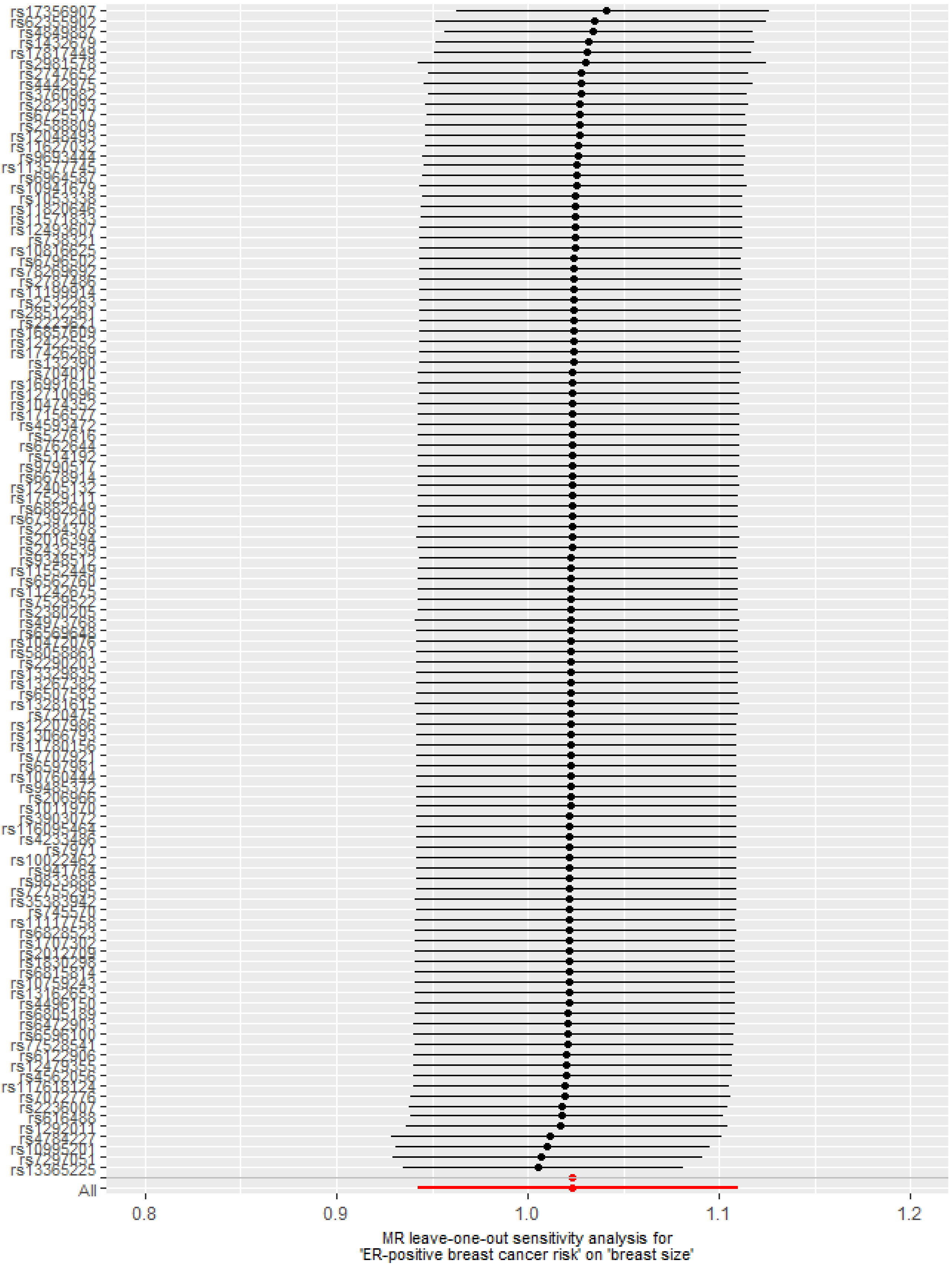
All
0.8
0.9
1.0
1.1
1.2
MR leave-one-out sensitivity analysis for
'ER-positive breast cancer risk' on 'breast size'

(F)

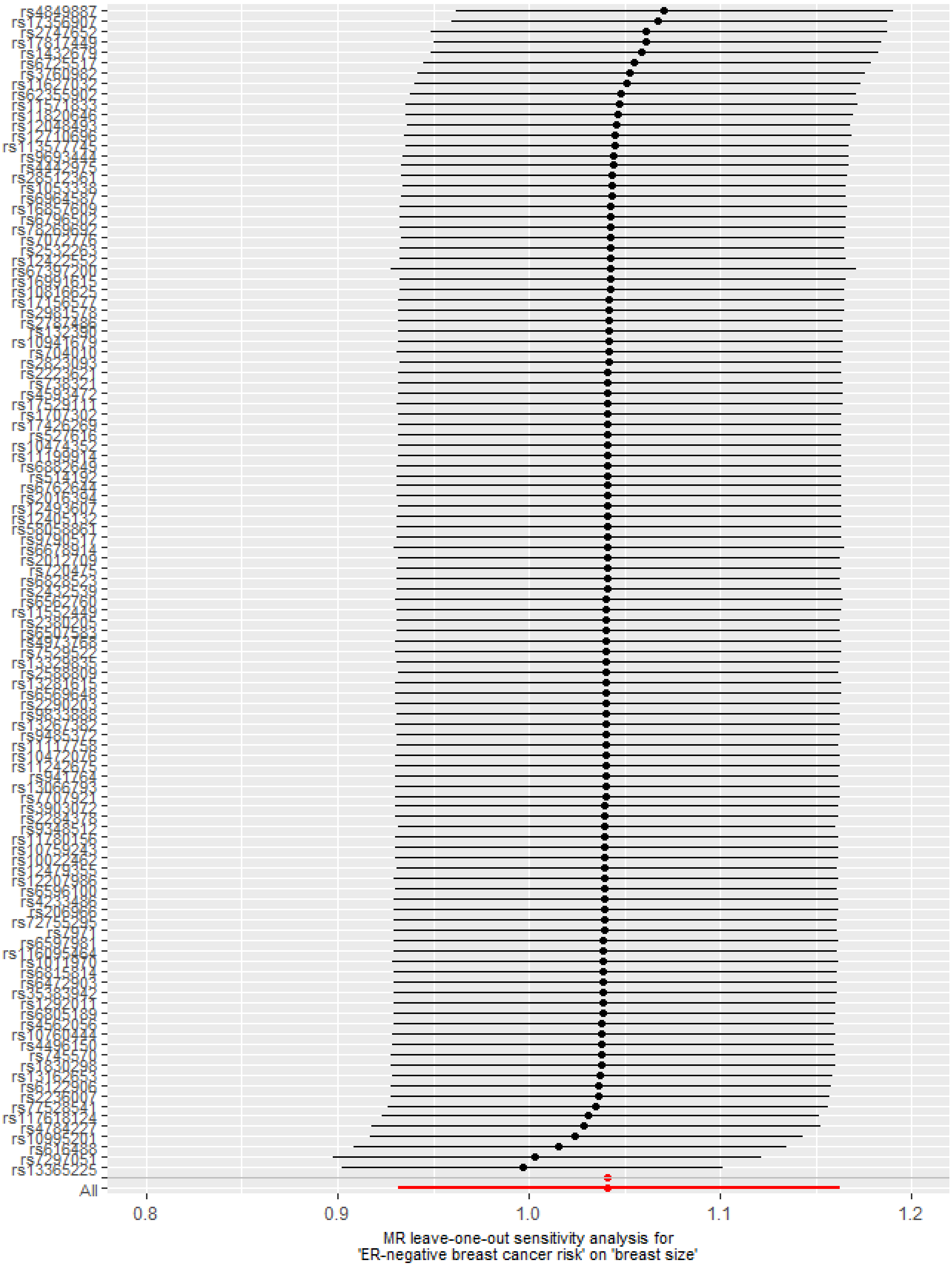
All
0.8
0.9
1.0
1.1
1.2
MR leave-one-out sensitivity analysis for
'ER-negative breast cancer risk' on 'breast size'
